# Intrinsic Electrocatalytic Activity for Oxygen Evolution of Crystalline 3d‐Transition Metal Layered Double Hydroxides

**DOI:** 10.1002/anie.202100631

**Published:** 2021-05-26

**Authors:** Fabio Dionigi, Jing Zhu, Zhenhua Zeng, Thomas Merzdorf, Hannes Sarodnik, Manuel Gliech, Lujin Pan, Wei‐Xue Li, Jeffrey Greeley, Peter Strasser

**Affiliations:** ^1^ The Electrochemical Energy, Catalysis, and Materials Science Laboratory Department of Chemistry Chemical Engineering Division Technical University Berlin Strasse des 17. Juni 124 10623 Berlin Germany; ^2^ School of Chemistry and Materials Science CAS Excellence Center for Nanoscience Hefei National Laboratory for Physical Sciences at Microscale University of Science and Technology of China Hefei 230026 Anhui China; ^3^ Davidson School of Chemical Engineering Purdue University West Lafayette IN 47907 USA

**Keywords:** electrochemical surface area, hydrothermal synthesis, layered double hydroxides, oxygen evolution reaction, water splitting

## Abstract

Layered double hydroxides (LDHs) are among the most active and studied catalysts for the oxygen evolution reaction (OER) in alkaline electrolytes. However, previous studies have generally either focused on a small number of LDHs, applied synthetic routes with limited structural control, or used non‐intrinsic activity metrics, thus hampering the construction of consistent structure–activity‐relations. Herein, by employing new individually developed synthesis strategies with atomic structural control, we obtained a broad series of crystalline α‐M_A_(II)M_B_(III) LDH and β‐M_A_(OH)_2_ electrocatalysts (M_A_=Ni, Co, and M_B_=Co, Fe, Mn). We further derived their intrinsic activity through electrochemical active surface area normalization, yielding the trend NiFe LDH > CoFe LDH > Fe‐free Co‐containing catalysts > Fe‐Co‐free Ni‐based catalysts. Our theoretical reactivity analysis revealed that these intrinsic activity trends originate from the dual‐metal‐site nature of the reaction centers, which lead to composition‐dependent synergies and diverse scaling relationships that may be used to design catalysts with improved performance.

## Graphical abstract

Catalytic activities for oxygen evolution on crystalline 3d transition metal layered double hydroxides are derived using electrochemical surface area based normalization. Density functional calculations reveal a dual‐metal‐site feature of the reaction centers that provides opportunities to design new catalysts with improved performance.

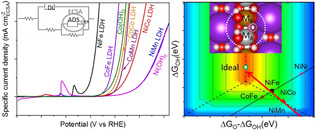

## Introduction

The electrochemical generation of hydrogen from the splitting of water is an established process to convert electricity into the chemical energy of hydrogen, a fuel with high gravimetric energy density. Water splitting electrolyzers are commercially available, and in these devices the hydrogen evolution reaction (HER) at the cathode is accompanied by the oxygen evolution reaction (OER) at the anode. One of the major conversion efficiency losses in electrolyzers is associated with the catalysis at the anode, where proton‐coupled multi‐electron transfers and multiple reaction intermediates result in slower kinetics than in the mechanistically simpler HER. In the acidic environment of polymer electrolyte membrane (PEM) electrolyzers, Ir‐based oxides represent the state of the art OER catalysts.^[1]^ In contrast, in neutral^[2]^ or in alkaline environments non‐precious metal‐based oxide catalysts, often based on 3d transition metals such as Mn, Fe, Co, and Ni, are the catalysts of choice.^[3]^ Systematic comparative studies with variable compositions and atomic structures, as well as the development of systematic benchmarking protocols, have, in turn, provided useful strategies to sift through the vast number of OER catalysts in non‐acid electrolytes.

There have been several such systematic studies, both experimentally and theoretically,^[4]^ since the pioneering work on OER catalysis by Rüetschi and Delahay in 1955,^[5]^ Bockris and Otagawa,^[6]^ and Trasatti^[7]^ in the 80s. In alkaline environments, the focus of comparative studies has been primarily placed on electrodeposited thin film oxide catalysts. One OER benchmarking study was conducted by Jaramillo and co‐workers, comparing selected electrodeposited Ni‐ and Co‐based metal oxide catalysts and an IrO_x_ standard.^[8]^ Other comparative studies focused on transition metal hydroxides and oxyhydroxides (in the following generally indicated as (oxy)hydroxides),^[9]^ which often form by surface reconstruction or amorphization on the surface of metal oxide catalysts in alkaline electrolytes.^[10]^ Boettcher and co‐workers investigated a series of electrodeposited (oxy)hydroxides in alkaline electrolyte (1 M KOH), where purification of the electrolyte to eliminate trace amounts of Fe impurities was performed for non‐Fe based catalysts.^[11]^ Their reported activity trend was: Ni(Fe)O_*x*_H_*y*_ > Co(Fe)O_*x*_H_*y*_ > FeO_*x*_H_*y*_‐AuO_*x*_ > FeO_*x*_H_*y*_ > CoO_*x*_H_*y*_ > NiO_*x*_H_*y*_ ≈ NiMnO_*x*_H_*y*_ > MnO_*x*_H_*y*_. These results highlighted the critical role of Fe in enhancing Ni and Co based (oxy)hydroxide catalyst activity, in agreement with early work by Corrigan.^[12]^ More recently, Chung et al. suggested that Fe dissolution and electrochemical re‐deposition over 3d transition‐metal hydr(oxy)oxide clusters yields dynamically stable Fe active sites, further underlining the importance of Fe for highly OER active and stable oxyhydroxide catalysts in alkaline environments.^[13]^

While the established reactivity trends of electrodeposited metal oxide catalysts in alkaline environments are compelling, a firm correlation of reactivity with their local or long range atomic structure has remained elusive. This is because electrodeposited metal hydroxide OER catalysts tend to possess low crystallinity, which is why they are referred to as XRD‐amorphous, and why advanced X‐ray characterization techniques are necessary to clarify their individual local atomic structures and correlate them with their activity.^[14]^ Well defined long‐range ordered metal (oxy)hydroxides phases can be prepared by solvothermal homogeneous precipitation methods,^[15]^ and their formation can be validated by X‐ray diffractometry. However, for the purpose of a comparative study, the synthesis procedure of a particular desired crystalline phase cannot be easily generalized and requires individual case‐by‐case adaption for all elemental combinations of the metal hydroxide ensembles. This is why previous studies focused either on a Ni‐ or a Co‐based hydroxide series, where synthetic protocols are better transferable.^[16]^ In contrast, high throughput screening studies^[17]^ investigating oxides and hydroxides beyond binary metal compositions were also reported.^[18]^ While high throughput methods rapidly collect data across a large pool of elemental compositions, they rely on standardized routine synthesis routes, and therefore often fail to achieve synthetic control over the crystal phase and structure of the catalysts.

In combination with experiments on structurally‐selected catalysts, DFT calculations permit understanding of their reactivity trends. On the one hand, advanced designing and screening methods have been developed to identify OER catalysts with superior activity. These methods include scaling‐based and scaling‐free optimization,^[19]^ the electrochemical step symmetry index,^[20]^ overpotential dependent volcano plots^[21]^ and overpotential‐dependent reaction free energies,^[22]^ among others.^[23]^ On the other hand, the active phases and catalytic mechanisms are still under debate even for classic OER catalysts such as IrO_*x*_.^[24]^ For alkaline OER on transition metal layered double hydroxides (LDH), apart from the promoting effects of Fe in NiFe and CoFe LDHs,[^15^, ^25^] a general understanding has not been reached. For example, it is unclear whether the OER trends that were established on those two most active catalysts can be extended upon introduction of a different element into the same hosts. It is also unknown whether those advanced designing and screening methods, which were developed on the basis of diverse model systems, are applicable to more specific and more realistic OER catalysts. Such a fundamental understanding is not only important for providing scientific insights to rationalize experimental trends, but is also crucial for establishing a more complete picture of OER mechanisms that can be used to calibrate the above designing methods and theories. These achievements would permit feasible design of OER catalysts with performance beyond the state‐of‐the‐art catalysts.

As previously discussed, current understanding of reactivity trends of metal (oxy)hydroxide OER catalysts in alkaline environments considers the presence of Fe as a necessary, yet not sufficient, criterion for high OER catalytic activities. In particular, NiFe and CoFe (oxy)hydroxides are generally reported to be the most active catalysts in the various activity trends reported in the literature, regardless of the strategy of normalization of the activity metrics (see a summary in Supplementary Table 1).[^8^, ^11^, ^13^, ^16b^] This is because they have much higher intrinsic activity than the other compounds. However, apart from these top catalysts, significant differences can be found in the order of the remaining catalysts; for example, contradictory reactivity orders were observed for NiMn and NiCo (oxy)hydroxides/LDHs versus Ni and Co (oxy)hydroxides (see a summary in Supplementary Table 1).[^8^, ^11^, ^13^, ^16b^] These and similar discrepancies are most likely caused by three factors: 1) different crystal phases and catalytically active centers between the catalysts, despite similar overall elemental compositions, 2) use of non‐intrinsic activity metrics or different approximations to the intrinsic activity, which may depend on experimental protocols and methodology, and 3) presence of unintentional impurities altering the surface kinetics, as previously pointed out in the case of Fe. While broad trends studies have been reported for XRD‐amorphous materials, those for well‐defined (oxy)hydroxide crystalline phases with long‐range order are scarce. Also, the existing activity trend studies have largely considered application‐relevant activity metrics, such as OER overpotentials at fixed current densities or, for instance, catalytic lower‐limit turn‐over‐frequencies based on total metal content rather than on surface area.

In this work, we seek to fill some of the above knowledge gaps by combining well‐defined experiments with rigorous calculations to study the intrinsic OER activities of well‐defined crystalline layered double hydroxides (LDH) across the group of late 3d transition metals in alkaline conditions. In comparison to previous studies,^[11]^ we synthesized crystalline LDH nanoplatelets, instead of XRD‐amorphous (oxy)hydroxide thin films, using new individually developed synthesis strategies with atomic structural control based on co‐ and homogeneous precipitation instead of cathodic electrodeposition. In addition, we derived their intrinsic activity through electrochemical surface area (ECSA) normalization, instead of total metal‐based turnover frequencies. In comparison to our previous publication,^[15]^ which identified and elucidated the catalytically active phase, the reaction center, and the OER mechanism of the most active NiFe LDH and CoFe LDH catalysts, this work systematically studies the intrinsic OER activity of a series of crystalline M_A_M_B_ LDHs, providing a fundamental understanding of the synergy (both positive and negative) between the dual metal atom sites that form the reaction centers, as suggested by our DFT calculations. Our study is hence characterized by: 1) unique preparation protocols with individually adjusted synthesis recipes that offered unprecedented control over the crystallinity and long range order of the desired alpha phase for both the Ni‐ and the Co‐ based LDH series; 2) the comparison of surface‐normalized intrinsic activity based on a very recently proposed advanced evaluation method of the electrochemical surface area of oxyhydroxide surfaces^[26]^ (this ECSA normalization is important, since a higher surface area can provide a higher number of OER active surface sites, resulting in a higher activity, despite a lower intrinsic activity of the sites); 3) electrolyte purification prior to the electrochemical experiments; and 4) theoretical insights that point to a design space of catalysts with OER performance beyond what is inferred from idealized scaling relationships.[^4a^, ^19^, ^23^, ^27^]

## Results

### Preparation and physico‐chemical characterization

**Figure 1:**
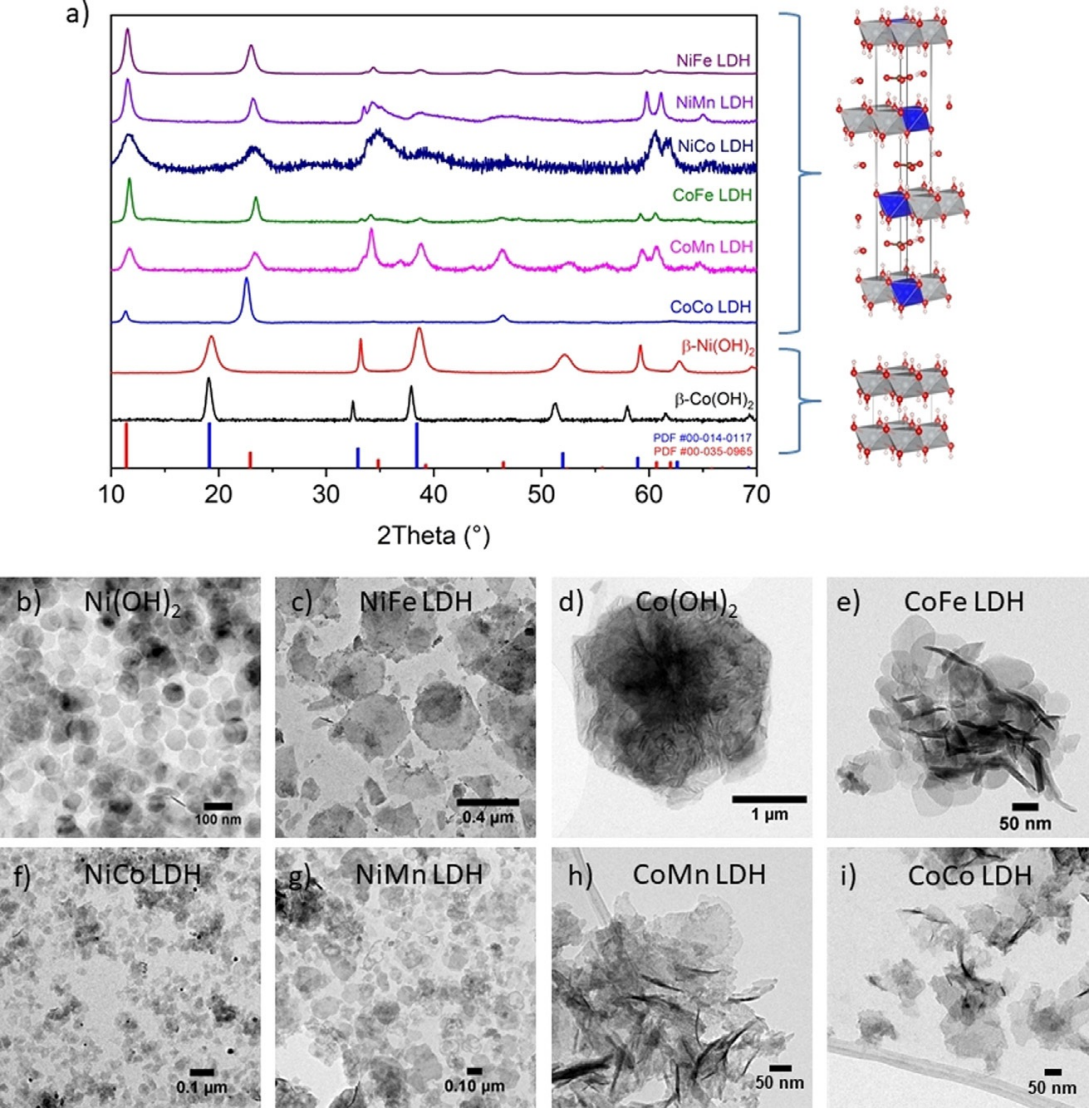
a) Normalized X‐ray diffraction patterns of the synthesized M_A_^II^M_B_^III^ LDHs and brucite‐like β‐Ni(OH)_2_ and β‐Co(OH)_2_. b)–i) TEM images showing the nanoplate morphology and sizes of the as‐prepared catalysts. For X‐ray diffraction patterns, examples for the corresponding crystalline structures are shown on the right. In the models, M_A_^II^ atoms are shown in gray, M_B_^III^ in blue, O in red, H in white, C in the carbonate anions in bronze. Note that carbonate is chosen as the representative anion intercalated within the LDHs in the model, though Co^II^Co^III^ LDH actually incorporates Br^−^. The reference patterns for hydrotalcite (red, PDF# 00‐035‐0965) and brucite‐like β‐Ni(OH)_2_ (blue, PDF# 00‐014‐0117) are also shown.

NiFe, NiCo, NiMn, CoFe, Co^II^Co^III^, CoMn layered double hydroxides (LDH) and β‐Ni(OH)_2_ and β‐Co(OH)_2_ were synthesized by specific wet chemistry methods that led to the desired crystallized phases, hydrotalcite‐like for the LDHs and brucite‐like for the two reference β‐hydroxides. The LDH catalysts can be grouped into two series, a Ni series and a Co series, by describing the LDH formation as the result of the incorporation of a second metal with oxidation state +3 in Ni^II^(OH)_2_ and in Co^II^(OH)_2_, respectively. Anions are intercalated in the layers of LDHs, compensating the positive charge created by the substitution of M_A_^II^ cations with M_B_^III^, and water molecules also occupy the interlayer region. In contrast, the brucite‐like phases do not have species intercalated between the layers. The XRD patterns, shown in Figure 1 a, confirmed that the targeted crystal phases were obtained. To achieve this, synthesis conditions were optimized specifically for each catalyst (see Supplementary Figure 1,2 and related discussion in supporting information for details). While the presence of <1–2 nm nanoparticles sized below coherently scattering domains could not be observed using microscopic techniques, it cannot be generally excluded. Nonetheless, the dominant LDH XRD peak reflections evidence the incorporation of the second metal (Fe, Co and Mn) in the Ni(OH)_2_ or Co(OH)_2_ hosts for all samples. To verify the LDH formation further, the metal oxidation states were estimated by the analysis of the X‐ray absorption near‐edge structure (XANES) (Supplementary Figure 3 and Supplementary Table 2), and found fully consistent with the expected M_A_^II^M_B_^III^ LDH chemical formula. The oxidation states for Co and Mn in CoMn LDH, 2.6 and 3.4 respectively, appeared slightly higher than expected, which is attributed to an excessive oxidation and deprotonation due to the hydrogen peroxide used in the synthesis. The selected metal compositions included in the present trend study are listed in Table 1 and determined by inductively coupled optical emission spectroscopy (ICP‐OES). For hetero‐metals LDHs, a 3:1 ratio of the host metal to the dopant metal was targeted, which falls in the range where maximum activity is observed for NiFe and CoFe oxyhydroxides.^[28]^ The only small variation was necessary for NiCo LDH, with a ratio of 2:1, for the phase purity requirement explained above. The morphology was investigated by transmission electron microscopy (TEM) and consistent with the expected nanoplate shape for these layered materials (Figure 1 b). The nanoplates showed large roughened terrace planes and thin edges, with dimensions that were found to vary strongly between the different catalysts (Figure 1 b and Supplementary Table 3).

**Table 1:** Elemental compositions by ICP‐OES.^[a]^

Samples	Atomic content		Metal weight percentage		
	M_A_/M_B_ Ratio	M_B_ (at %)	M_A_ (wt %)	M_B_ (wt %)	Total metal (wt %)
NiFe LDH	3.55 (3:1)	22	43.41	10.17	53.58
NiMn LDH	2.94 (3:1)	25	45.03	14.33	59.36
NiCo LDH	1.79 (2:1)	36	42.42	24.12	66.54
CoFe LDH	3.30 (3:1)	23	35.51	10.76	46.27
CoMn LDH	3.30 (3:1)	23	38.17	11.54	50.36
Co^II^Co^III^ LDH	–	–	–	–	32.84
Ni(OH)_2_	–	–	–	–	78.45
Co(OH)_2_	–	–	–	–	69.30

[a] Targeted elemental ratios are given in brackets. The higher metal weight % in the two brucite‐like catalysts is due to the absence of intercalated water and anions.

### Surface redox chemistry and OER overpotentials

**Figure 2:**
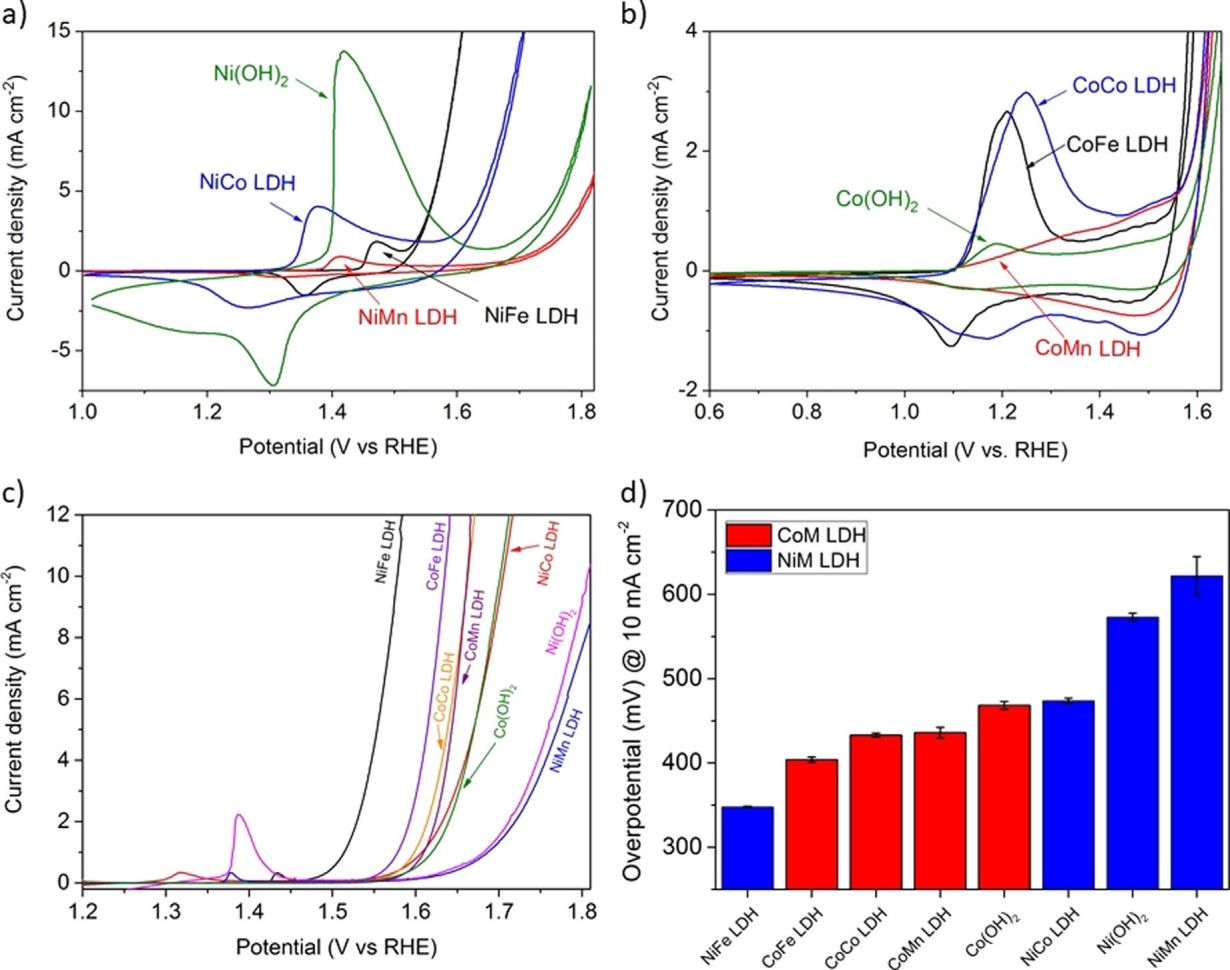
Cyclic voltammetric curves for the Ni series (a) and the Co series (b). The 10th cycle of the cyclic voltammetric activation treatment is shown. Scan rate: 50 mV s^−1^. Linear sweep voltammetry curves recorded at 1 mV s^−1^ (c) and corresponding OER overpotentials at 10 mA cm^−2^ (d). In (d), red is for the Co series and blue for the Ni series. Error bars represent the standard deviations of the averaged values of multiple samples. Curves in (c) for NiFe LDH, CoFe LDH, Ni(OH)_2_ and Co(OH)_2_ and corresponding data in (d) have been reproduced from Ref. [15].

Each member of the prepared array of OER electrocatalysts was subsequently tested using rotating disk electrodes (RDEs) in 0.1 M KOH, depositing the same catalyst loading of 0.1 mg cm^−2^ on glassy carbon cylinders. The electrolyte was purified of Fe traces according to published methods, using sacrificial Ni(OH)_2_ and Co(OH)_2_ for the Ni^II^M_B_ and Co^II^M_B_ LDH series, respectively.[^28a^, ^29^] An activation treatment consisting of cycling the potential from the resting potentials (≤1 V_RHE_) to OER relevant potentials is first applied. During this treatment, the peaks in the cyclic voltammetry associated with the surface metal redox chemistry grew in intensity, which is attributed to more sites becoming electrochemically accessible. The potential cycling also causes structural and electronic transformations from hydroxides to oxyhydroxides, which can be reversible, for NiFe LDH, or only partially reversible, for CoFe LDH.^[15]^ In Figure 2 a and b, the 10^th^ cycles of each catalyst are compared. These catalysts showed the rich surface redox chemistry of the component 3d transition metals. The characteristic Ni^II^ oxidation peak and the corresponding reduction peak are shown in Figure 2 a at potential lower than the OER onset. The Ni oxidation peak is shifted more anodically for NiFe LDH, more cathodically for NiCo LDH, and remains roughly unshifted for NiMn LDH in respect to Ni(OH)_2_. These shifts indicate that an electronic effect is affecting the stability of Ni centers in the +2 oxidation state in the various LDHs, which is consistent with incorporation of the second metal. The area of the peaks also changes significantly, with Ni(OH)_2_ showing the highest peak. This increase may be mainly caused by the fact that it has the highest surface area among all samples synthesized in the current work, as quantified in the next section, and partially related to a larger amount of Ni atoms in Ni(OH)_2_ than in the other LDHs. Figure 2 b shows the cyclic voltammetric curves for the Co series, where double oxidation peaks and corresponding reduction peaks are observed, which is typical of Co‐based (oxy)hydroxides and attributed to Co^II^ and Co^III^ oxidation. Potential shifts of the oxidation peaks were small in comparison to the Ni series and difficult to evaluate, partly due to the broadness of the peaks. The less anodic redox couples, assigned to oxidation/reduction of Co^II^, appear considerably suppressed and slightly anodic shifted for CoMn LDH in respect to the other catalysts. The same peaks in the CV of CoFe LDH do not show appreciable anodic shifts with respect to Co(OH)_2_, as elsewhere reported for electrodeposited CoFe oxyhydroxides, where the shift became particularly large at above 30 Fe at %.^[28a]^ This might be due to relatively low Fe at % in the CoFe LDH. Co(OH)_2_ showed the smallest peak areas, which is most likely related to the low surface area, as will be discussed further below.

After the cyclic voltammetric activation, linear sweep voltammetry (LSV) at the slow scan rate of 1 mV s^−1^ was performed to evaluate the OER activity (Figure 2 c). The OER overpotentials evaluated at 10 mA cm^−2^ are shown in Figure 2 d. Fe‐containing NiFe and CoFe LDHs lead the activity trend and are followed by the other Co containing catalysts. Ni(OH)_2_ and NiMn LDH occupy the lowest positions in the trend with the highest overpotentials. While the positive synergy between M‐Fe is clear in Figure 2 d and well documented in the literature, the synergy between M‐Mn is ambiguous in both Figure 2 d and in literature. For NiMn and NiCo, we observed a neutral or slightly negative synergy based on overpotential comparison, which is consistent with the results reported by Markovic, Boettcher and co‐workers,[^11^, ^13^] but in contrast to the positive synergy reported by Jaramillo, Calle‐Vallejo and co‐workers.[^8^, ^16b^] For Co‐Mn, we observed a surprising result, with a positive synergy in comparison with Co(OH)_2_, consistent with results reported by Hu and co‐workers,^[30]^ but a slightly negative synergy in comparison with CoCo LDH. Such a conclusion can be more clearly established at a lower current density, 1 mA cm^−2^ (Supplementary Figure 4,5). Below, we will use ECSA‐based intrinsic activity to reconcile these apparent discrepancies.

### ECSA determination and OER intrinsic activity

The overpotential at a specified current density is a convenient metric for comparing the OER activity of the investigated catalysts, since it is related to the potential losses during operation, which is important for the commercial application of the catalysts in electrolyzers. However, this metric convolutes several parameters, and a lower overpotential might be obtained by an increase in the number of active sites or by the presence of a different specific site with higher reactivity. Turn over frequencies (TOF) are calculated to estimate the intrinsic activity of catalysts.^[31]^ However, for LDHs catalysts, the nature of the active sites is often unknown, and their surface concentration is also difficult to calculate. In our case, the roughening of the edges of the nanoplates (Figure 1 b–i) and the possible partial exfoliations^[32]^ of part of the terraces of the nanoplates, typical of layered materials, complicate the application of geometric models to estimate the surface concentration of active sites. Therefore, “bulk” TOF, considering all the metal centers, are often calculated and indicated as a lower limit of the TOF (Supplementary Figure 6). However, this approach limits their effectiveness in providing real intrinsic activity trends.

**Figure 3:**
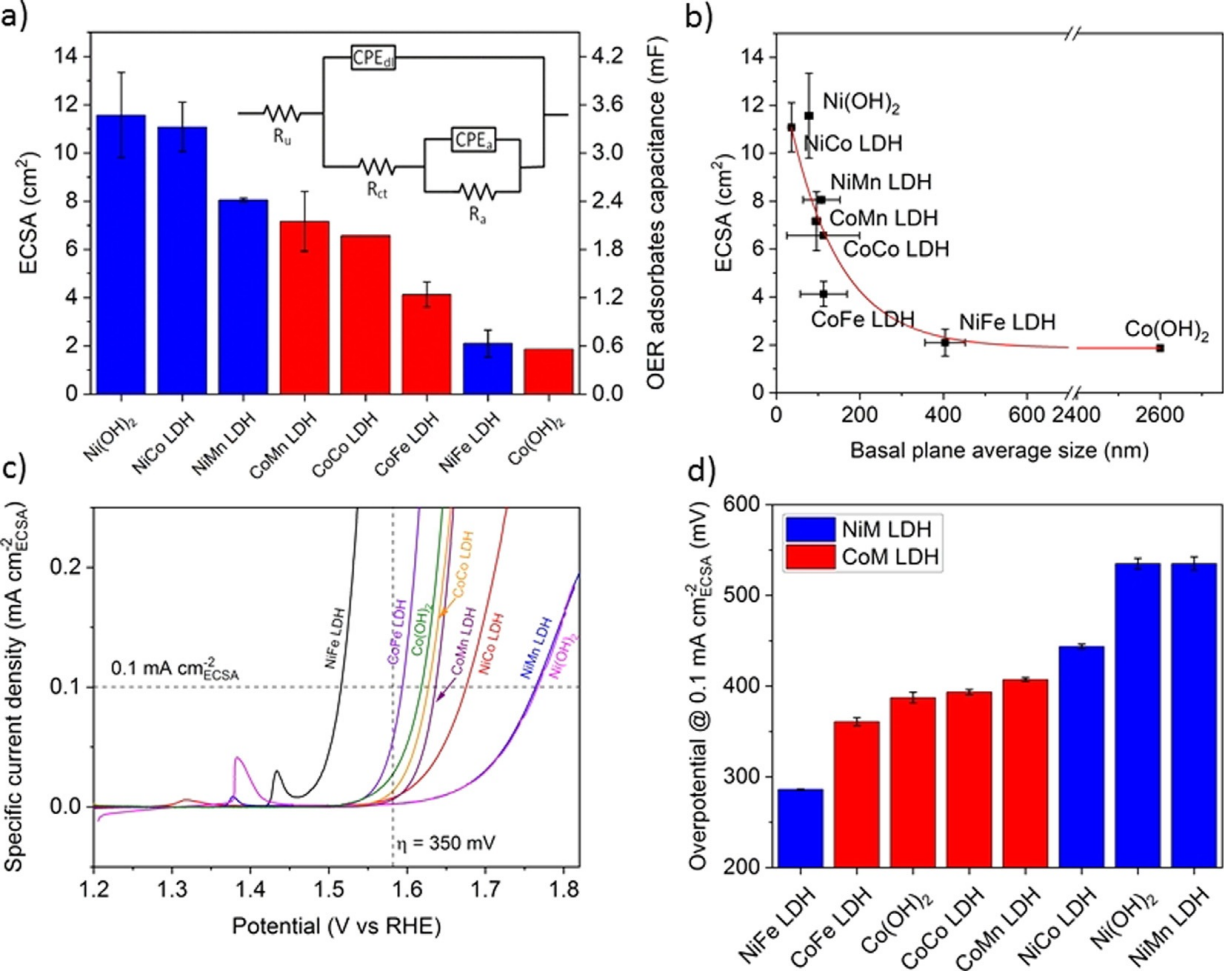
ECSA obtained by EIS method for the LDH and brucite‐like catalysts (a). The ECSA is calculated from the CPE_a_ after dividing by the specific area capacitance (*C*_s_) of 0.3 mF cm^−2^. The inset in (a) shows the equivalent circuit scheme used to fit the impedance data. The indexes of the resistances R and the constant phase elements CPE stand for: u: uncompensated, ct: charge transfer (Faradaic process), dl: double layer and a: adsorbates (OER intermediates). ECSA trend in relation to the average basal plane size of the nanoplates (b). The error bars represent standard deviations and the red line is a guide to the eyes. The large size reported for Co(OH)_2_ is probably the results of strong agglomerations. Linear sweep voltammetry curves recorded at 1 mV s^−1^ showing the current densities normalized by the ECSA values (c). Dashed horizontal and vertical lines show the values of 0.1 mA cm^−2^_ECSA_ and the overpotential of 350 mV, which are used to evaluate overpotentials, as in (d), and specific activities, as in Supporting Information Figure S7 c), respectively. d) OER overpotentials at 0.1 mA cm^−2^_ECSA_. In (a) and (d) red is for the Co series and blue for the Ni series. Error bars in (a) and (d) represent the standard deviations of the averaged values of multiple samples.

Specific activities obtained by normalizing the current to the electrochemically active surface area (ECSA) can be used as alternative methods if the TOFs cannot be accurately calculated. For LDHs, the evaluation of the ECSA is not straightforward, due to 1) potential dependent changes of the electrical conductivity of LDHs,^[33]^ 2) a narrow or non‐existing potential window that is free of faradaic current in the conductive regime, 3) metal oxidation peaks in cyclic voltammograms that overlap with the OER faradaic current, and 4) difficulties in obtaining model catalysts with smooth planar surfaces for the conversion of the calculated values, that is, capacitances, in the unit of an area.^[34]^ Among the various methods to estimate the ECSA, we used the capacitance of the adsorbed OER intermediates (*C*_a_) that was calculated by electrochemical impedance spectroscopy (EIS) at 1.6 V_RHE_. This potential is more anodic than the oxidation peak associated to the oxidation of Ni^II^ and Co^II^, in which NiM and CoM LDH become conductive.^[11]^ The equivalent circuit which was used to fit the impedance data was adapted from Watzele and Bandarenka,^[26]^ and previously introduced and discussed by Lyons and Brandon.^[35]^ The value *C*_a_ was calculated from the associated constant phase element and the parallel resistance (Figure 3 a). The specific unit area capacitance (*C*_S_) of 0.3 mF cm^−2^ that was obtained from a smooth Ni(OH)_2_ surface by Watzele and Bandarenka was used to convert the *C*_a_ capacitances into real surface areas.^[26]^ Ideally, for each material, a specific capacitance obtained by a smooth unitary surface of the same material should be used. However, because of the difficulty of obtaining smooth surfaces for metal hydroxide catalysts, specific capacitances for surfaces of equal roughness factor and similar (few nanometer) height variation as for Ni(OH)_2_ are not currently available. It is expected that deviations of specific capacitance among these catalysts would be small, however, due to the similarity in their crystal structure. Thus, the value from Ni(OH)_2_ was used for all catalysts, as was assumed for the specific double layer capacitance in metal oxides.^[8]^ Under this assumption, this method for determining the ECSA for transition metal LDH electrocatalysts solves and overcomes all the mentioned issues.

Figure 3 a shows the ECSA of the catalysts. Interestingly, NiFe LDH and Co(OH)_2_, which have the largest lateral sizes (Figure 1 b–i and Supplementary Table 3) have the lowest ECSAs, while Ni(OH)_2_ and NiCo LDH, which have the smallest lateral nanoplate sizes, have the highest ECSA (Figure 3 b). This behavior supports the validity of the calculated ECSAs. We will show that while those differences do not influence the order of the most and least active catalysts, they have a substantial influence on those with intermediate activities, that is, Fe‐free Co‐containing LDHs. Such an influence has a profound impact on the reconciliation of discrepancies in literature and on the development of a fundamental understanding of synergy between M_A_‐M_B_. The ECSA‐normalized current densities, and the derived trend in overpotentials, is shown in Figure 3 c,d (the specific current density trend at the overpotential of 350 mV is also shown in Supplementary Figure 7). NiFe LDH is the most active catalyst, followed by CoFe LDH. Ni(OH)_2_ and NiMn LDH are the least active. This trend implies a neutral or slightly negative synergy between Ni‐Mn, which reconciles the discrepancies in literature.[^8^, ^11^, ^16b^] Interestingly, due to the large differences in ECSA, the activity of CoCo LDH and Co(OH)_2_ are now comparable. This suggests that similar active sites are present in both catalysts during OER and that the presence of Br^−^ anions in the as prepared CoCo LDH has a negligible effect (within error bar) on the activity. However, operando structural studies are necessary for definitive conclusions. Furthermore, the specific activity trend showed that the intrinsic activity of these two Co catalysts is superior with respect to NiCo LDH, which showed one of the highest ECSAs. This conclusion is in contrast to the other activity metrics that do not account for the active surface area. Thus, for Fe‐free Co‐containing species, the orders are changed from CoCo LDH, CoMn LDH, Co(OH)_2_, NiCo LDH in the geometric‐area based overpotential trend to Co(OH)_2_, CoCo LDH, CoMn LDH, NiCo LDH in the ECSA‐normalized trend. The most notable change is that there is a negative synergy between Ni‐Co and Co‐Mn based on the intrinsic activity, while a neutral or positive synergy was derived based on the overpotentials in the current work and in some literature.[^8^, ^30^] Thus, to reconcile those discrepancies in literature and achieve a fundamental understanding of synergy between M_A_‐M_B_, normalization using ECSA is essential. Otherwise, contradictory conclusions may be drawn for a catalyst with the same composition but different ECSA due to different synthesis methods. This may be also the reason for some other discrepancies in literature. Below, we will further study these synergies (both positive and negative) between M_A_‐M_B_ through DFT calculations.

The proposed intrinsic activity trend for crystalline transition metal LDHs is also important for the discussion about the effect of disorder on the activity for NiFe oxyhydroxides and, in general, for this type of catalysts.^[36]^ While a trend for XRD‐amorphous transition metal (oxy)hydroxides was provided by Boettcher and co‐workers,^[11]^ there was no comparable intrinsic activity trend to date for the corresponding crystalline LDH catalysts. Furthermore, in contrast to an all metals‐based TOF, which provides reasonable estimate for intrinsic activity only for very thin films,^[11]^ the ECSA‐based method is well suited for both amorphous and crystalline catalysts, allowing the comparison of their activity using the same method in future works.

### Revealing composition‐dependent synergies through DFT calculations

**Figure 4:**
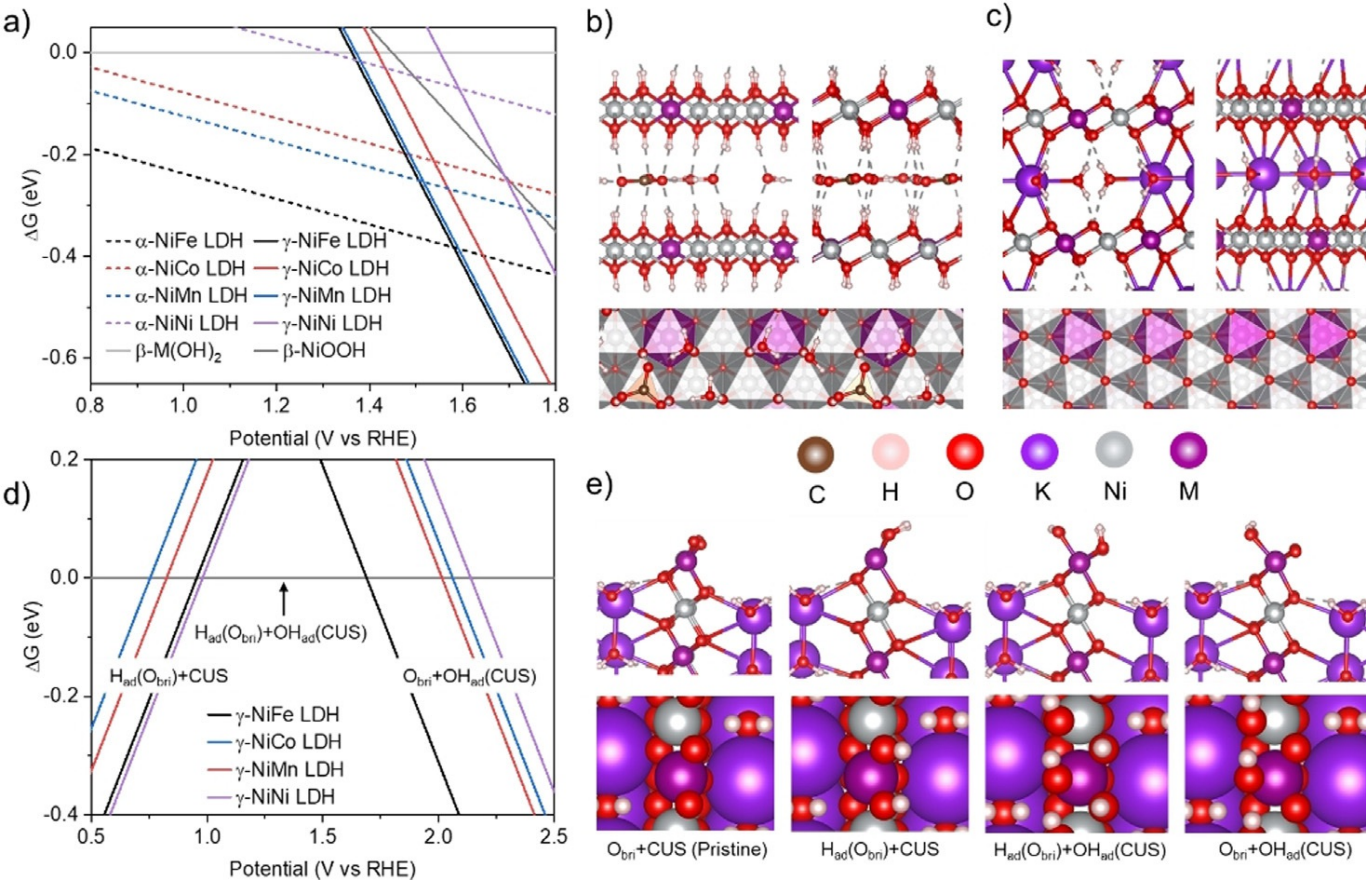
a) Free energy diagram of α‐ and γ‐NiM LDH (M=Fe, Co, Ni, Mn) with respective to the anhydrous oxides β‐M(OH)_2_. Front, side, and top views of the α‐NiM LDH (b) and γ‐NiM LDH (c). d) Surface free energy diagram of γ‐NiM LDH with respective to the surface saturated with hydrogen adsorption (H_ad_) on bridge O (O_bri_) and OH adsorption (OH_ad_) on coordinatively unsaturated site (CUS), that is, H_ad_(O_bri_)+OH_ad_(CUS). e) Front and top views of pristine surface O_bri_+CUS that does not exist under electrochemical environments, H_ad_(O_bri_)+CUS at the low potential, H_ad_(O_bri_)+OH_ad_(CUS) at the intermediate potential, and O_bri_+OH_ad_(CUS) at the high potential.

The atomic‐scale structures of the as‐prepared phase and the active phase of NiFe and CoFe LDHs, the α‐phase and the γ‐phase with water molecules and ions intercalated between layers, have been identified in our previous work.^[15]^ Here, using those structures as the starting point, we studied the stability of M_A_M_B_ LDHs synthesized in the current work through DFT calculations (Supplementary Table 4); additional details of the DFT calculations are given in the computational methods section in the supporting information. The bulk phase diagrams indicate that the phase transitions from α‐NiM LDH (Figure 4 a, b) and α‐CoM LDH (Supplementary Figure 8) are around 1.5 V and 1.2 V, respectively, which are consistent with the redox peaks in CV curves (Figure 2). These results suggest that γ‐phases (Figure 4 c) are the active phases for OER. Their geometric structures and electronic structures, including magnetic moments, are summarized in Supplementary Figure 9 and Table 5–9.

To facilitate comparison with our experimental results, we focus on the NiM series which has a larger span of the activity than that of CoM series, and we used (01–10) surfaces that are exposed at the edge of the γ‐NiM LDH sheets to study their OER performance. The electrolyte was implicitly included in the calculations through the solvation corrections (Supplementary Table 10), which were evaluated in our previous work through ab initio molecular dynamics simulations with explicit liquid water filling of the vacuum region between the model and the image.^[15]^ We first evaluated catalysts’ steady‐state surface structure through surface free energy diagrams (Figure 4 d,e). We found that, under OER conditions, the surface bridge oxygen (O_bri_) sites are saturated with H adsorption (H_ad_) by forming bridge OH* (“*” indicates a coordinatively unsaturated surface site or vacancy), and the coordinatively unsaturated metal sites (CUSs) are saturated by OH adsorption (OH*). Thus, OER on these surfaces starts from the deprotonation of the surface OH* species (OH* → O*), the so‐called Mars van Krevelen mechanism. We considered the following four consecutives steps for the OER:

(1) $$\mathrm{OH}{}^{-}+\mathrm{OH}{}^{*}\to O{}^{*}+H{}_{2}O+e{}^{-}$$

(2) $$\mathrm{OH}{}^{-}+O{}^{*}\to \mathrm{OOH}{}^{*}+e{}^{-}$$

(3) $$\mathrm{OH}{}^{-}+\mathrm{OOH}{}^{*}\to O{}_{2}+H{}_{2}O+e{}^{-}+{}^{*}$$

(4) $$\mathrm{OH}{}^{-}+{}^{*}\to \mathrm{OH}{}^{*}+e{}^{-}$$

**Figure 5:**
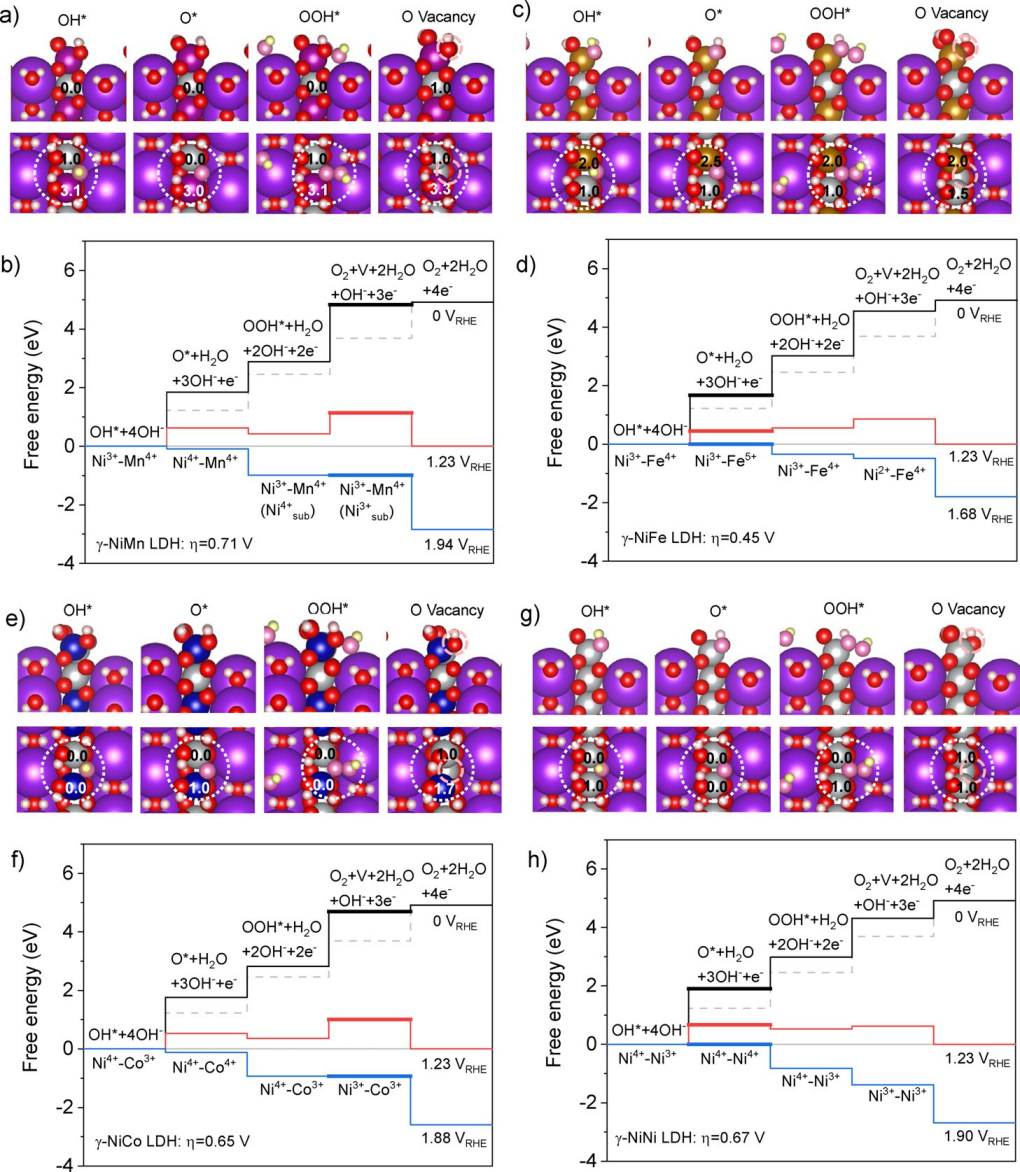
Structures of reaction intermediates (a, c, e, g) and reaction free energy diagrams (b, d, f, h) for OER on γ‐NiM LDH (M=Mn, Fe, Co, or Ni). The color schedule of atoms is the same as that in Figure 4, except for M (purple for Mn in (a), brown for Fe in (b) and navy for Co in (c)) and the reaction intermediates (yellow is used instead of white for hydrogen and rose instead of red for oxygen). A dashed rose circle indicates the formation of a surface O vacancy. The reaction free energies at 0 V are in black, 1.23 V red, and the potential when the potential‐determining steps become downhill in blue. Ideal steps of 1.23 eV are shown with dashed gray lines. The potential limiting steps are highlighted with thick lines. The numbers on the atoms at the reaction centers are their magnetic moments in the Bohr magneton (μ_B_). The oxidation state, which is deduced based on the intrinsic magnetic moments (see Supporting Information Table S5–S9), are given in the reaction free energy diagram.

The corresponding reaction free energies are denoted as Δ*G*_1_(*U*), Δ*G*_2_(*U*), Δ*G*_3_(*U*) and Δ*G*_4_(*U*), respectively. *U* is the electrode potential in the reversible hydrogen electrode (RHE) scale. We note that this four‐step reaction pathway has been corroborated by the spectroscopic identification of the OOH* intermediates on Au‐ and Co‐based catalysts.^[37]^ The calculated reaction free energies indicate that, consistently with our previous work, the oxidation of two‐metal coordinated bridge OH* moieties is more favorable than that of one‐metal coordinated atop OH*. Effectively, the O‐bridged M_A_‐M_B_ dual metal site, instead of M_A_ or M_B_ single metal site, is the reaction center. The maximum reaction free energies at 1.23 V_RHE_, max(Δ*G_n_*(1.23 V_RHE_), *n*=1, 2, 3 or 4), are 0.45 eV, 0.48 eV, 0.65 eV, 0.67 eV and 0.71 eV for γ‐NiFe, γ‐CoFe, γ‐NiCo, γ‐NiNi, and γ‐NiMn LDH (Figure 5 and Supplementary Table 11). Thus, to make those reaction free energies downhill, overpotentials (*η*) of 0.45 V, 0.48 V, 0.65 V, 0.67 V and 0.71 V (*η*=(Δ*G* −1.23 eV)/e), respectively, need to be applied. The trend of the calculated overpotentials is consistent with the trend of overpotentials evaluated at 0.1 mA cm^−2^ from ECSA‐normalized current densities, except for NiMn (Figure 3 d).

For γ‐NiMn LDH, the calculated overpotential implies a slightly negative synergy between Ni and Mn. As a consequence, the measured activity may stem primarily from Ni‐Ni, instead of Ni‐Mn reaction centers, due to their higher activity. For γ‐NiCo, though the calculated overpotential is slightly smaller than that of γ‐NiNi, it is larger than that of the Co‐Co reaction centers analyzed in our previous work (e.g. 0.6 V).^[15]^ Thus, while there is a positive synergy between Ni and Fe, and Co and Fe,^[15]^ our study suggests a negative synergy between Ni and Co, and Ni and Mn. Below, we will demonstrate that these composition‐dependent synergies originate from the unique geometric structures and electronic structures of the dual‐metal‐site reaction centers. Those unique features have important implications for breaking scaling relationships and enlarging the design space of oxyhydroxide catalysts with OER activity beyond the state‐of‐the‐art catalysts.

### Revealing diverse OH‐O scaling relationships at dual‐metal‐site reaction centers

**Figure 6:**
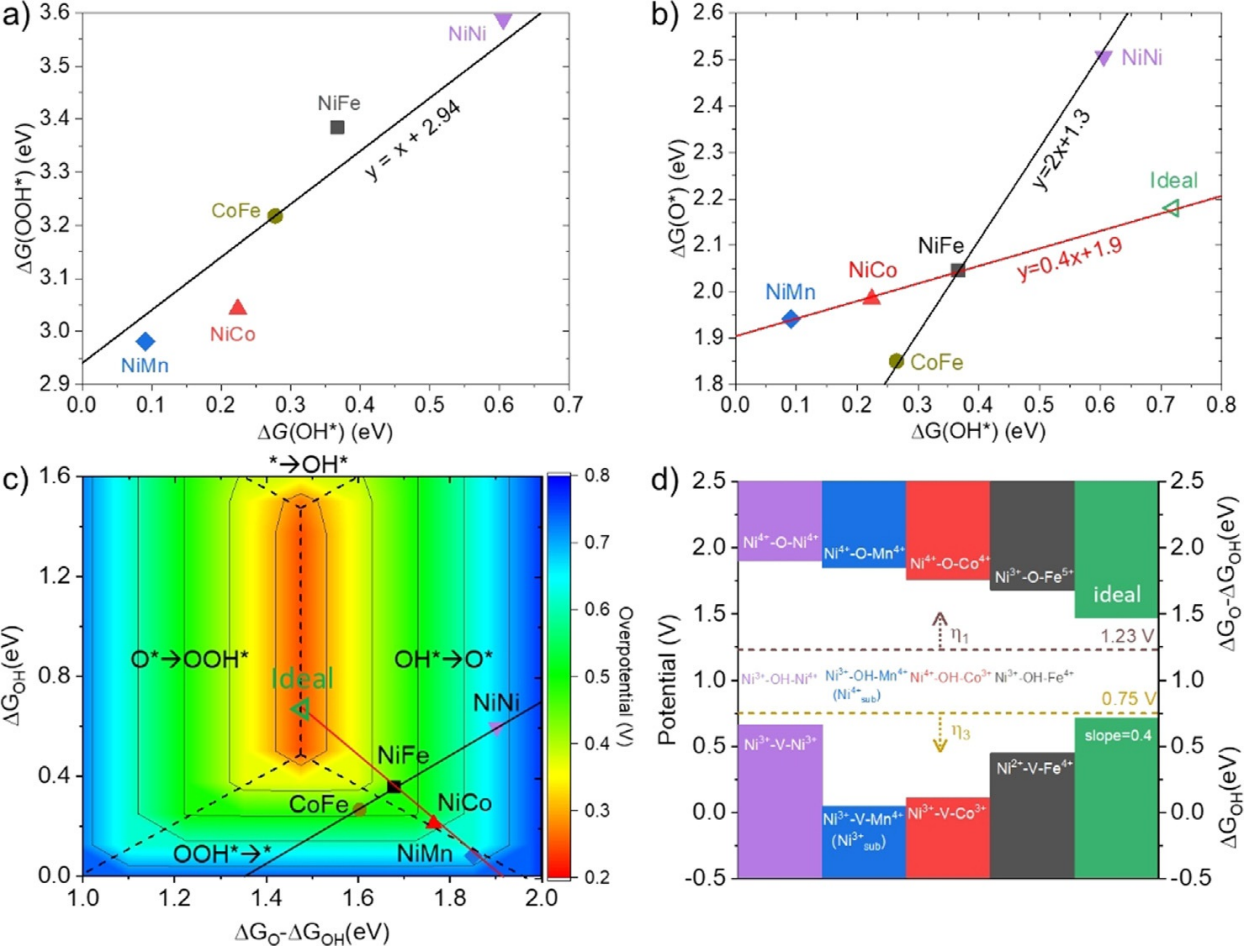
a) Scaling relationship of OH* and OOH* intermediates at the reaction centers (OH‐OOH scaling relationship). b) Scaling relationship of OH* and O* intermediates at the reaction centers. The lines are fitted using three points. c) 2D volcano of OER overpotential, which is constructed based on the OH‐OOH scaling relationship in the current work; the reaction energy of OH*→O*→OOH* is 2.94 eV. Such a scaling leads to a value of 1.98 eV in the reaction energy of OOH*→O_2_+*→OH* steps ((1.23*4)−2.94=1.98). Herein, we chose the OH*→O* step and *→OH* step as the two independent descriptors, for the *x*‐axis and *y*‐axis of the 2D volcano, respectively. The solid lines are based on the scaling relationships fitted in (b) using three points. The dashed lines separate the zones of overpotential that were determined by different reaction steps. d) The correlation of electronic structures and overpotentials of the O‐bridged Ni‐M (M=Mn, Fe, Co and Ni) reactive centers with respect to two consecutive steps of O oxidation, OH adsorption (V→OH*) and OH oxidation (OH*→O*). Electronic structures are represented by the oxidation state of the metals in the reaction centers. Horizontal dashed lines indicate the potentials at which there is no overpotential for the two potential limiting steps, OH*→O* step (1.23 V) and OOH*→O_2_ +* step (0.75 V). The differences between the colored bars and those dashed lines represent the corresponding overpotentials, η_1_ and η_3_, for the two steps, respectively. “Ideal” denotes an ideal catalyst, that is, one on top of the volcano. “V” stands for a vacancy site.

Figure 6 shows the OH‐OOH and OH‐O scaling relationships (the corresponding values are reported in Supplementary Table 12). For the OH‐OOH scaling relationship, we obtained a slope of one and an intercept of 2.94 (Figure 6 a). It is worth noting that, for the intercept, the values of 3.0±0.2 eV were reported in the literature, with a dependence on the functional used in the calculations.[^4a^, ^38^] The intercept in the current work is nearly identical to those obtained with the other dispersion corrected functionals.^[38]^ However, the OH‐O scaling relationship has a much more complex behavior than that of OH‐OOH. While NiNi, NiFe and CoFe follow the ideal slope of 2,^[27]^ NiCo and NiMn significantly deviate from this trend, resulting in actual slopes that generally vary from 0.4 to 2 (Figure 6 b). There are also cases of negative slopes (CoFe‐NiCo and CoFe‐NiMn), which break the ideal scaling relationship entirely. We note that due to the limited set of data points used for the calculation of the slopes and their small differences in energy, for example, about 50 meV for some cases, our work demonstrates the plausible existence of diverse slopes, rather than the specific values of these slopes. The diversity of slopes is inherently rooted in the unique thermodynamics of each individual element, which follow the general trends only when included with a set of elements with large energy span, on the scale of 5 eV.[^4a^, ^27^, ^39^] In the literature, those deviations and possibilities of diverse slopes were usually treated as part of the error hidden behind the general trends. As the absolute errors are usually on the order of 0.2 eV, and some individual deviations are 0.5 eV or larger,[^27^, ^38a^, ^39^] disentangling the errors and deviations may significantly enlarge the design space for catalysts, as we propose in this work.

The scaling relationships and their implications for catalyst design are illustrated in the plot of the 2D activity volcano (Figure 6 c). The top of the volcano is determined by the OH*→O*→OOH* steps and so is limited by the OH‐OOH scaling relationship. In contrast, the above predicted overpotentials for the γ‐LDHs are determined either by the OH*→O* step or by the OOH*→ O_2_+* step. This is also shown in Figure 6 c, where the data points are either in the zone of OH*→O* or OOH*→O_2_+ * as the potential limiting step. It is worth noting that, for the cases with OOH*→O_2_+* as the potential limiting step, the enrichment of OOH* on the surface makes its experimental measurement feasible. This is likely the reason for the experimental observation of OOH intermediates on Au‐based and Co‐based catalysts.^[37]^ As the top of the volcano is at the intersection of the OH*→O* step and the O*→OOH* step, not of any other two steps, the identification of the OH*→O* and OOH*→O_2_+* steps as the potential‐limiting steps implies that the minimum overpotential that is determined by the intersection of these two steps is away from the top of the volcano. Therefore, even if the systems follow the ideal scaling relationships (e.g. OH‐O scaling relationship with slope of 2), there would be no catalysts that could even approach the optimal activity dictated by the OH‐OOH scaling relationship. This is visualized in Figure 6 c, where the line representing the ideal OH‐O scaling relation does not cross the top of the volcano. Below, we will demonstrate that, while OH‐OOH scaling relationships determine the tops of volcano curves, the OH‐O scaling relationships determine whether it is possible to approach them. We will show that the dual‐site nature of the reaction centers in the LDHs studied in the current work provides the possibility of breaking the OH‐O scaling relationship and enlarges the design space of oxyhydroxide catalysts with OER activity beyond the state‐of‐the‐art catalysts.

To understand the origin of diverse scaling relationships and its implications, we performed electronic structure analyses of the reaction centers under steady state conditions and in response to the adsorption of OER intermediates (Figure 5 and Figure 6 d). Under OER conditions, for γ‐NiOOH (γ‐NiNi LDH), the atoms at the reaction center are O‐bridged Ni^3+^‐Ni^4+^ (the oxidation states are determined on the basis of the intrinsic magnetic moment of each element, along with considerations of the charge balance—see Supplementary Table 5–9 and computational methods). For NiM LDHs, Fe and Mn atoms occupy Ni^4+^ sites by forming O‐bridged Ni^3+^‐Fe^4+^ and Ni^3+^‐Mn^4+^ reaction centers, while Co atoms occupy Ni^3+^ sites by forming an O‐bridged Co^3+^‐Ni^4+^ reaction center. Based on electronic structure analyses (Figure 5 and Figure 6 d), three major features can be derived for the redox of the reaction centers during the OER.

First, although a reaction center is formed by two metal atoms, only one of them plays a major role in each reaction step of the OER. For example, for γ‐NiOOH with an O‐bridged Ni^3+^‐Ni^4+^ reaction center, 3+ sites play a major role on the OH*→O* step, as evidenced by the accompanying Ni^3+^→Ni^4+^ and the intact oxidation state of Ni^4+^ site (Figure 5 and Figure 6 d). On the other hand, the 4+ sites of Ni^3+^‐Ni^4+^ reaction center play a major role in the OOH*→O_2_+* step, as evidenced by the accompanying Ni^4+^→Ni^3+^ and the intact oxidation state of Ni^3+^ sites (Figure 5 and 6 d). For NiCo LDH with the Co^3+^‐Ni^4+^ reaction center, similar to that of gamma‐NiOOH, 3+ sites play a major role on the OH*→O* step, and 4+ sites play a major role on the OOH*→O_2_+* step (Figure 5 and Figure 6 d).

Second, despite of the above similarity between O‐bridged Ni^3+^‐Ni^4+^ and Co^3+^‐Ni^4+^ reaction centers, metal sites that are primarily involved in each reaction step cannot be deduced a priori. For example, for NiFe with the Ni^3+^‐Fe^4+^ reaction center, 4+ sites, instead of 3+ sites, play a major role on the OH*→O* step, as evidenced by the accompanying Fe^4+^→Fe^5+^, while 3+ sites, instead of 4+ sites, play a major role on the OOH*→O_2_+* step, as evidenced by the accompanying Ni^3+^→Ni^2+^ (Figure 5). We note that, as OH*→O* is the potential limiting step, the formation of transient Fe^5+^ from the oxidation of Fe^4+^ occurring during the OER might not be directly observable in conventional experiments. On the other hand, the Fe^4+^ oxidation state, which is present under steady state conditions, has been observed through operando Mössbauer spectroscopy.^[40]^

Third, in addition to dual metal atoms that form the reaction centers and are in contact with the reaction intermediates, third atoms that are not in direct contact with the reaction intermediates also can be involved in the reaction. For example, for NiMn LDH with the Ni^3+^‐Mn^4+^ reaction center, subsurface Ni^4+^ sites, instead of either surface Ni^3+^ or surface Mn^4+^, play a major role on the OOH*→O_2_+* step, as evidenced by the accompanying Ni_sub_^4+^→Ni_sub_^3+^ transition (Figure 5).

Thus, it is the dual‐metal‐site feature of the reaction centers, the composition‐dependent involvement of the metal sites, and the possible involvement of a third site that allow OER on LDHs to deviate from the ideal OH‐O scaling relationship. To further understand the OH‐O scaling relationship in LDHs, Figure 6 d summarizes the features of OH and O adsorption on the reaction centers. The OH adsorption on the vacancies (V) of the Ni^3+^‐V‐Ni^3+^, Ni^3+^‐V‐Mn^4+^‐(Ni^3+^_sub_), Ni^3+^‐V‐Co^3+^and Ni^2+^‐V‐Fe^4+^ reaction centers leads to the oxidation of one surface or subsurface site (i.e. Ni^3+^, Ni^3+^_sub_, Ni^3+^ and Ni^2+^, respectively), as well as the formation of Ni^3+^‐Ni^4+^, Ni^3+^‐Mn^4+^, Co^3+^‐Ni^4+^ and Ni^3+^‐Fe^4+^ reaction centers (Figure 5 and Figure 6 d). The O adsorption leads to the oxidation of two sites, either two surface sites or one surface site and one subsurface site (Figure 5 and Figure 6 d). Due to the different chemical nature of those two sites, their average oxidation energy likely deviates from the oxidation energy of each individual site, which leads to the deviation from the ideal scaling relationship. For O adsorption, which can be considered as two consecutive steps, OH adsorption and OH oxidation, that occur on two different sites, the deviation from the ideal scaling relationship implies that it may be possible to disentangle and tailor the energetics of each individual step. Such a tunability provides a design space of catalysts with OER performance (Figure 6 c and d) beyond what is constrained by the ideal scaling relationships.

For example, based on the nearly ideal OH‐O scaling relationship of NiNi‐NiFe‐CoFe, the OER activity can be improved only by slightly decreasing the binding energy of the OH* intermediate and the reaction energy of the OH*→O* step, in comparison with that on NiFe. This is because decreasing the binding energy of the OH* intermediate leads to, on the one hand, a decreased overpotential for the OH*→O* step, but on the other hand, it causes an increased overpotential for the OOH*→O_2_+* step. When the overpotentials of those two steps become identical, further decreasing the binding energy of the OH* intermediate would make OOH*→O_2_+* the potential limiting step, resulting in an increased overpotential, as with the case of CoFe LDH. In contrast, the OH‐O scaling relationship of NiFe‐NiCo‐NiMn with a slope of 0.4 could lead to a decreased overpotential on both the OH*→O* step and the OOH*→O_2_+* step, reaching the minimum dictated by the OH‐OOH scaling relationship by weakening the OH adsorption energy by 0.25 eV in comparison with that on NiFe (see Figure 6 b–d). The corresponding reduction of the overpotential implies an improvement in the OER activity of over three orders of magnitude.

As OH* and OH*→O* are two independent descriptors that uniquely determine the overpotential in the 2D volcano, Figure 6 d also provides a facile way to predict the OER overpotential, and potentially sheds light on possible combinations of reaction centers with further improved activity. We note that other strategies and descriptors have been proposed for the design of OER catalysts with significantly improved performance, such as the scaling‐based and scaling‐free optimization,^[19]^ the electrochemical step symmetry index,^[20]^ the overpotential dependent volcano plot,^[21]^ and the overpotential‐dependent reaction free energy,^[22]^ among others.^[23]^ To design new catalysts with the activity that is up to three orders of magnitude higher than that of the state‐of‐the‐start catalysts, however, we propose that a possible route is to break the OH‐O scaling relationship by forming binary metal oxyhydroxides with dual metal sites at the reaction centers, or by introducing a third element into NiFe or CoFe LDHs.

## Conclusion

By combining well‐defined experiments with rigorous calculations, we conducted a systematic analysis of the intrinsic OER activity and composition‐dependent synergy of a comprehensive set of crystalline LDH catalysts prepared from late 3d transition metals (Ni, Co, Fe, Mn). A unique synthesis protocol was developed for each and every compositional combination to ensure the presence of a well‐defined atomic crystal structure, the alpha crystal phase, at the outset of the kinetic tests. All catalysts showed the characteristic nanoplatelet morphology. The intrinsic OER activity was obtained by normalization using the electrochemical surface area that was determined through the analysis of the adsorbed OER intermediate capacitance. Synergies (both positive and negative) arising from each specific binary metal LDH combination resulted in characteristic variations in the surface redox electrochemistry and the catalytic OER activity. The activity trend in terms of the overpotentials at fixed geometric electrode area‐based OER current densities revealed that Fe‐containing LDHs invariably displayed the highest kinetic OER activities, followed by Fe‐free Co‐based LDH catalysts, and finally by LDHs without Fe nor Co. Trends in intrinsic OER activities obtained by ECSA normalization confirmed this trend, which in its most generalized form reads: NiFe LDH ≫ CoFe LDH > Co(OH)_2_, CoCo LDH > NiCo LDH, CoMn LDH, ≫ Ni(OH)_2_, NiMn LDH. We found that the adopted ECSA normalization is critical to eliminate the effects of varying real surface areas on the activity and reconciles the discrepancies of overpotential based activity in literature. Also, unlike overpotential and total metal mass‐based activities, real surface‐area normalized OER activities have direct relevance for theoretical reactivity modelling. Our DFT calculations suggest that the above activity and the composition‐dependent synergy originates from the dual‐metal site feature of the reaction centers. While this feature does not influence OH‐OOH scaling relationship, it leads to diverse OH‐O scaling relationships, including those with near‐zero slopes and the negative slopes. This diversity provides a route to approach the top of volcano curve that is dictated by the OH‐OOH scaling relationship. Thus, catalysts with dual active sites provides a design space of catalysts with OER performance beyond what is constrained by the ideal scaling relationships.

## Conflict of interest

The authors declare no conflict of interest.

## Supporting information

As a service to our authors and readers, this journal provides supporting information supplied by the authors. Such materials are peer reviewed and may be re‐organized for online delivery, but are not copy‐edited or typeset. Technical support issues arising from supporting information (other than missing files) should be addressed to the authors.

Supplementary
