# Supplementary material for: Intrinsic Electrocatalytic Activity for Oxygen Evolution of Crystalline 3d‐Transition Metal Layered Double Hydroxides
Source: Angew Chem Int Ed Engl. 2021 May 26;60(26):14446–57. doi: 10.1002/anie.202100631 (PMC8252729; doi:10.1002/anie.202100631)
Supplement: Supplementary file 1 — Supplementary [file ANIE-60-14446-s001.pdf]

Angewandte Chemie
Eine Zeitschrift der Gesellschaft Deutscher Chemiker

Supporting Information

Intrinsic Electrocatalytic Activity for Oxygen Evolution of Crystalline 3d-Transition Metal Layered Double Hydroxides

Fabio Dionigi[+], Jing Zhu[+], Zhenhua Zeng,* Thomas Merzdorf, Hannes Sarodnik, Manuel Gliech, Lujin Pan, Wei-Xue Li, Jeffrey Greeley, and Peter Strasser*

anie_202100631_sm_miscellaneous_information.pdf

Table of Contents

Supplementary Figures **4**
Supplementary Figure 1. Optimized synthesis conditions for CoFe LDH and CoMn LDH. 4
Supplementary Figure 2. Optimized synthesis parameters for NiCo LDH and NiMn LDH. 5
Supplementary Figure 3. Metal-edge XANES. 6
Supplementary Figure 4. OER overpotentials at current density of 1 mA cm^{-2}. 6
Supplementary Figure 5. Tafel plots. 7
Supplementary Figure 6. Turn over frequencies based on metal content. 7
Supplementary Figure 7. ECSA normalized specific activity. 8
Supplementary Figure 8. Bulk phase diagrams of Co-based oxides. 8
Supplementary Figure 9. Geometric structures of LDHs. 9
Supplementary Tables **10**
Supplementary Table 1. Qualitatively comparison among selected OER activity trends in alkaline electrolytes for transition dual metal (oxy)hydroxide catalysts from the literature. 10
Supplementary Table 2. Co and Mn oxidation states from XANES analysis. 11
Supplementary Table 3. Average nanoplate sizes by TEM analysis. 11
Supplementary Table 4. Relative stability of LDHs. 12
Supplementary Table 5. Intrinsic magnetic moment of Mn, Fe, Co and Ni in MO, M_2O_3 and MO_2 with 2+, 3+ and 4+ oxidation state. 12
Supplementary Table 6. Atomic magnetic moments (μB) of α-M_AM_B LDHs. 12
Supplementary Table 7. Atomic magnetic moments (μB) of γ-M_AM_B LDHs. 13
Supplementary Table 8. Atomic magnetic moments (μ_B) of β-M_AM_B oxyhydroxides. 13
Supplementary Table 9. Atomic magnetic moments (μ_B) as a function of oxidation state. 13
Supplementary Table 10. Thermodynamic correction used in the free energy calculations. 14
Supplementary Table 11. Reaction free energy (eV) and overpotential η(V) on the γ-LDH surfaces. 14
Supplementary Table 12. Adsorption free energy of intermediates on the γ-LDH surfaces. 14
Experimental Methods **15**
Catalysts synthesis 15
Physico-chemical characterization 16
Ink and catalyst layer preparation 16
RDE measurements 16
Electrochemical protocol 17
ECSA calculation 18
TOF calculations 18
X-Ray absorption spectroscopy and calculation of oxidation states 19

Computational Methods **19**
Computational parameters 19
Thermodynamic correction 20
Computational details of α-phase and γ-phase LDHs 20
Determination of oxidation states 22
Supplementary Discussions **23**
Supplementary discussion on the synthesis of crystalline M_AM_B LDHs 23
Supplementary Discussion on Tafel Slopes 23
References **24**

Supplementary Figures

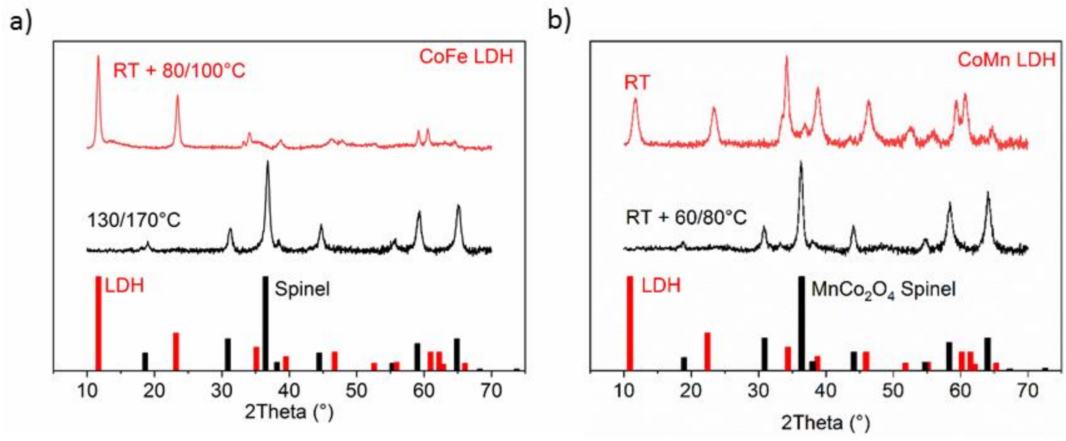

Supplementary Figure 1. Optimized synthesis conditions for CoFe LDH and CoMn LDH. (a) XRD pattern of CoFe LDH that was obtained with a coprecipitation step at room temperature (RT) in N_2 purged solution followed by a two-step solvothermal treatment at 80°C and 100°C (red) compared with the results of the application to Co and Fe precursors of the homogeneous precipitation method developed for NiFe LDH (i.e. two-step solvothermal synthesis in autoclave at 130°C and 170 °C, without preliminary coprecipitation) (black). The two patterns match the hydrotalcite MgAl LDH reference patterns (red, PDF #00-035-0965) and the Co_3O_4 spinel pattern (black, PDF #00-042-1467), respectively. (b) XRD patterns of CoMn LDH that was obtained with a coprecipitation step at room temperature (RT) in N_2 purged solution (red) compared with the results of the application of the two-step solvothermal treatment in autoclave at 60°C and 80 °C to the coprecipitated sample (black). The two patterns match the hydrotalcite MgAl LDH reference patterns (red, PDF #00-035-0965) and the $MnCo_2O_4$ spinel pattern (black, PDF #00-023-1237), respectively.

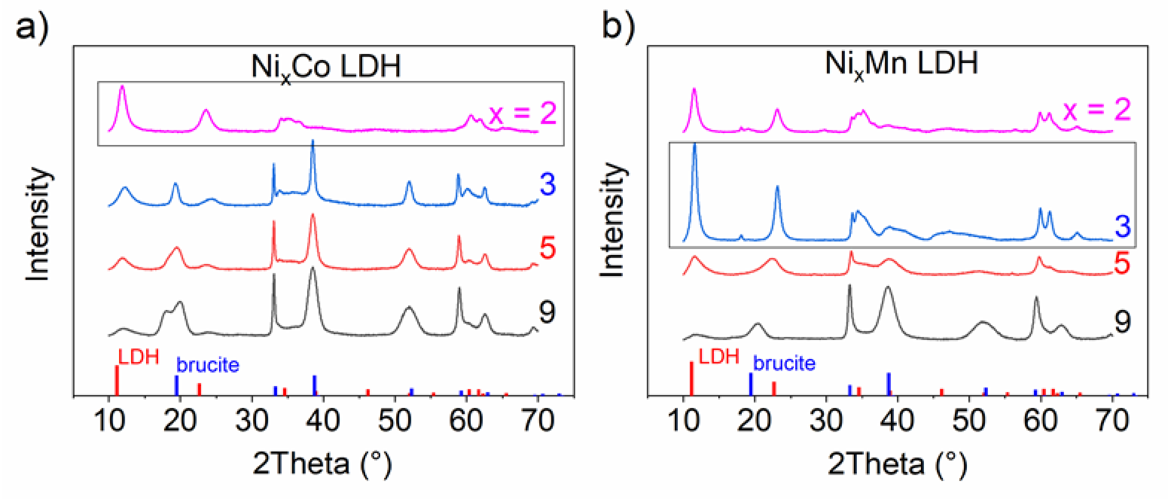

Supplementary Figure 2. Optimized synthesis parameters for NiCo LDH and NiMn LDH. XRD patterns for NiCo LDH obtained by different amount of Co (a). XRD patterns for NiMn LDH obtained by different amount of Mn (a). A square box highlights the XRD pattern corresponding to the selected catalyst for the OER activity study. The reference patterns for hydrotalcite (red, PDF# 00-035-0965) and brucite-like β-$Ni(OH)_2$ (blue, PDF# 00-014-0117) are also shown. For low amount of second metal content, a mixture of brucite and LDH phases is obtained. These phases do not guarantee the incorporation of the second metal (Mn or Co) into the $Ni(OH)_2$ host. Therefore, since one of the major aims of this study is to evaluate the electronic effect of the second metal on the OER activity, they were not further investigated. In Ni_xMn LDH with X=2 (b), the peaks at 2Theta angles of 18.2°, 29.9°, 35.3° 76.8° indicate the presence of a segregated Mn_3O_4 phase (PDF#00-013-0162, not shown). Among those reflections, only the peak at 18.2° is visible in NiMn LDH with X=3, suggesting that this impurity is also present in this catalyst, even though in less extent, and indicating that increasing the Mn content leads to the formation of this segregate phase.

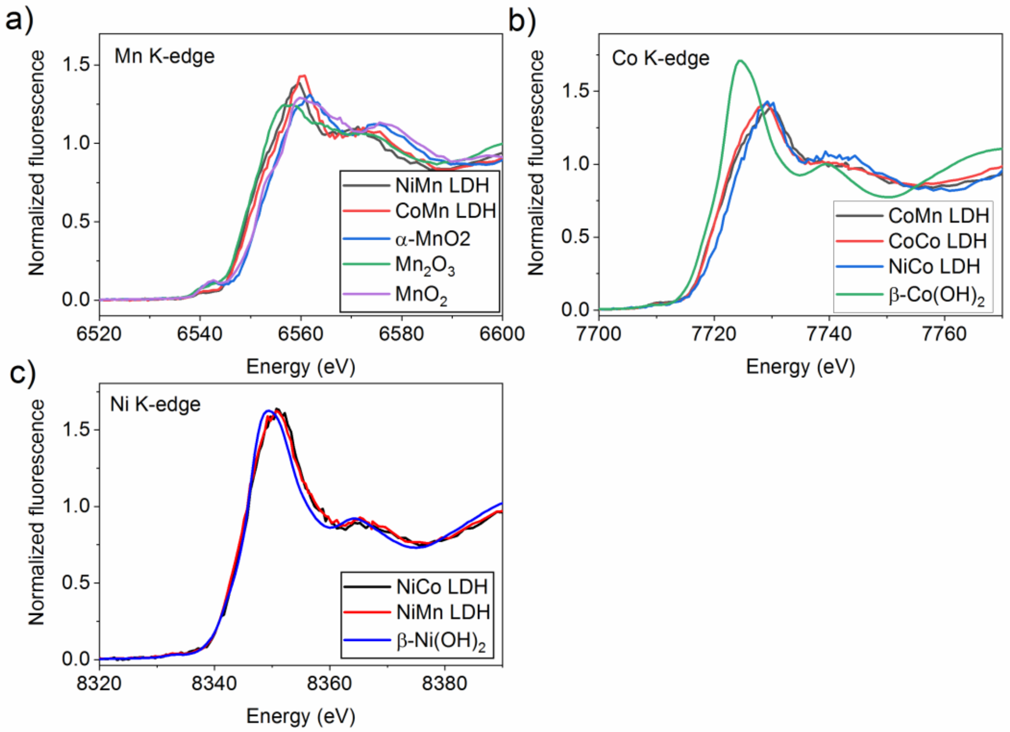

Supplementary Figure 3. Metal-edge XANES. Comparison of Mn K-edge (a), Co K-edge (b) and Ni K-edge (c) of NiMn LDH, CoMn LDH, CoCo LDH, NiCo LDH, β-$Ni(OH)_2$ and β-$Co(OH)_2$. α-MnO_2, Mn_2O_3 and MnO_2 were also measured as references for the Mn K-edge comparison.

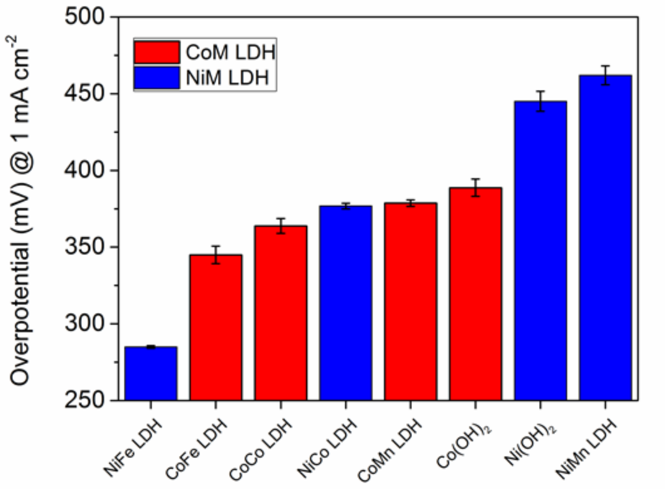

Supplementary Figure 4. OER overpotentials at current density of 1 mA cm^{-2}. OER overpotentials at 1 mA cm^{-2}. Red and blue colours have been used for the Co and Ni series, respectively. Error bars represent the standard deviations of the averaged values of multiple samples.

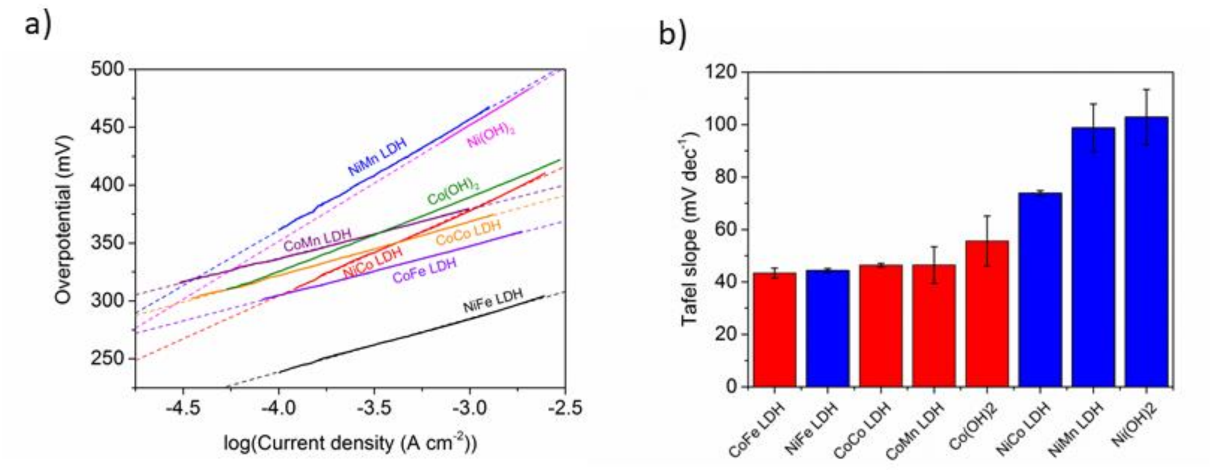

Supplementary Figure 5. Tafel plots. Tafel plots considering for each catalyst the low overpotential linear slope range (a) and corresponding Tafel slopes (b).

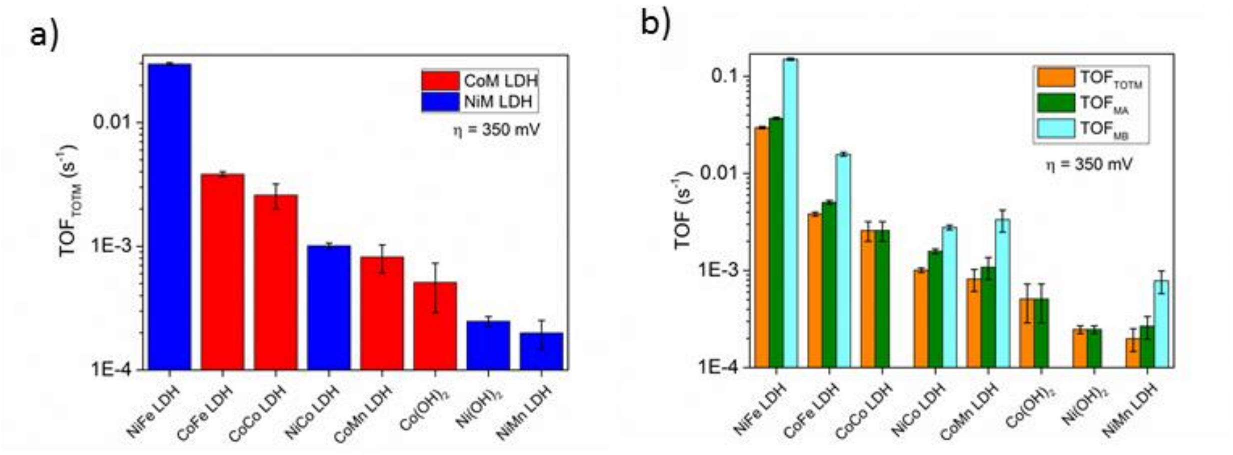

Supplementary Figure 6. Turn over frequencies based on metal content. Turn over frequency (TOF) calculated by normalizing the current at η = 350 mV by the total number of metal sites (TOTM) (a). In Panel (a), red and blue colours have been used for the Co and Ni series, respectively. The TOFs from panel (a) are reported in panel (b) (orange) and compared with the TOFs calculated by considering only metal M_A (green) and only metal M_B (cyan). Error bars represent error propagation form standard deviations of the averaged current densities obtained from multiple samples. The TOF_{TOTM} is a lower estimation of the real TOF, since it considers all the metal atoms on the GC as active sites. TOF_{MA} and TOF_{MB} represent the lower limit TOF in case the active sites consist only of M_A and M_B, respectively, since they still do not discriminate between bulk and surface sites. Additionally, this way of calculating the TOF does not discriminate between single metal O-μ_1 site or bridged O-μ_2 sites.

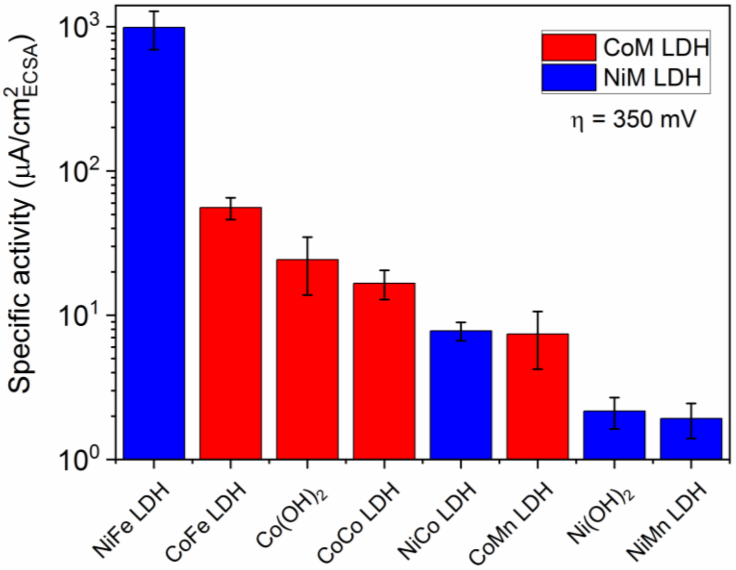

Supplementary Figure 7. ECSA normalized specific activity. Specific activity obtained by normalizing the current at η = 350 mV of LSV curves at 1 mV^{-1} by the ECSA. Red and blue colours have been used for the Co and Ni series, respectively. Error bars represent the propagation of the errors on ECSA and current at 350 mV, which were standard deviations of average values of multiple samples. Values for NiFe LDH and $Ni(OH)_2$ are reported with permissions from ref. [5].

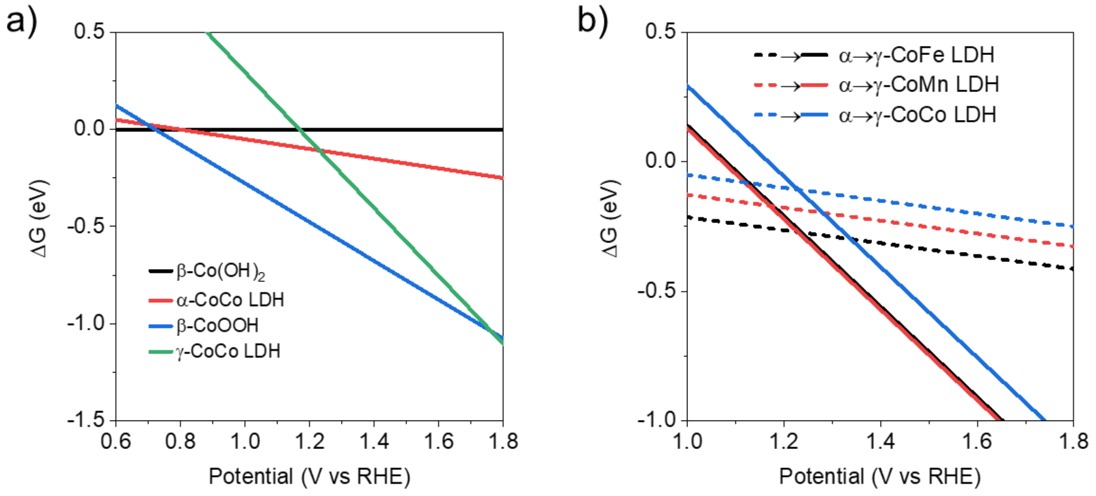

Supplementary Figure 8. Bulk phase diagrams of Co-based oxides. (a) Stability of pure Co phases (anhydrous oxides β-$Co(OH)_2$ and β-CoOOH, α-Co(II)Co(III) LDH, γ-Co(III)Co(IV) LDH). (b) Stability of α- and γ-CoM LDH (M = Fe, Mn, Co) with respective to the anhydrous oxides β-$Co(OH)_2$.

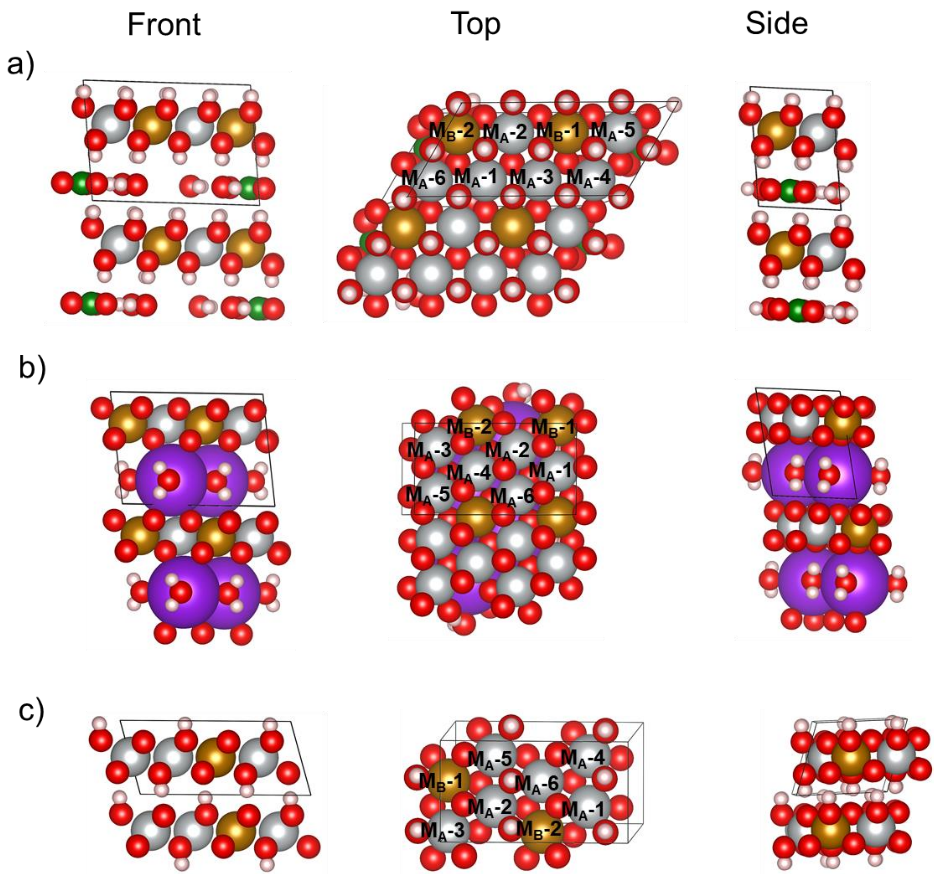

Supplementary Figure 9. Geometric structures of LDHs. The calculated unit cells of (a) α-M_AM_B LDHs, (b) γ-M_AM_B LDHs and (c) β-M_AM_B oxyhydroxides (M_A = Ni, Co, and M_B = Mn, Fe, Co, Ni) are indicated by the solid-line frames. Metal M_A and M_B, C, K, O and H are represented by silver, yellow, green, purple, red and white balls, respectively.

Supplementary Tables

Supplementary Table 1. Qualitatively comparison among selected OER activity trends in alkaline electrolytes for transition dual metal (oxy)hydroxide catalysts from the literature. The comparison is limited to selected works that reported trends among at least 4 catalysts. It includes also catalysts indicated as metal oxides in the original work, but that are synthesized with similar conditions as typically used for (oxy)hydroxides and might be expected to have an (oxy)hydroxides surface in alkaline electrolytes. The table highlights the different conditions used for obtaining the trends and the type of catalysts, including both intrinsic (ECSA-based methods) and non intrinsic (TOF total metals, overpotentials) activity metrics. A quantitatively comparison would require the same conditions. Note that the catalysts are not identical when comparing different works: despite belonging to the same large family of transition metal (oxy)hydroxides, they show for example different crystalline structure. To simplify the notation, only the metals are indicated, omitting O, H and intercalated species. CoCo stands specifically for CoCo LDH, while Co stands generically for CoO_xH_y. The table also lists if a further purification of the electrolyte (purif.) was reported in the original work. Comments concerning the general applicability of the different activity metrics are also provided. η_{OER} stands for the OER overpotential. C_{ads} and C_{DL} denotes the capacitance of OER adsorbate intermediates and diffusion layer, respectively. OCP stands for open circuit potential.

**Catalyst activity trend (and catalyst type)**	**Electr.**	**Activity metrics**	**Ref.**
NiFe >> CoFe > Co, CoCo > CoMn > NiCo >> Ni, NiMn<br><br>(crystalline LDH nanoplates)	0.1 M KOH (purif.)	η_{OER} @ 1 mA cm^{-2}_{ECSA}<br>(ECSA by EIS (C_{ads}) @ OER (1.6 V_{RHE}))*	This work
NiFe >> CoFe > Co, CoCo > NiCo, CoMn >> Ni, NiMn	“	ECSA-normalized current densities (ECSA by EIS (C_{ads}) @ OER (1.6 V_{RHE}))*	“
NiFe >> CoFe > CoCo, CoMn > Co, NiCo >> Ni > NiMn	“	η_{OER} @ 10 mA cm^{-2}	“
NiFe >> CoFe > CoCo > NiCo, CoMn > Co >> Ni > NiMn	“	η_{OER} @ 1 mA cm^{-2}	“
NiFe >> CoFe > CoCo > NiCo, CoMn, Co >> Ni, NiMn	“	TOF total metals<br>@ 350 mV	“
NiFe > CoFe > Fe > Co, NiCo > NiMn, Ni, NiCu<br><br>(electrodeposited metal clusters on Pt(111))	0.1 M KOH (purif.)	Geometric current densities at 1.7 V_{RHE}	[1]
Ni(Fe) > Co(Fe) > Fe/Au > Fe > Co > Ni ~ NiMn > Mn<br><br>(electrodeposited thin films)	1 M KOH (purif.)	TOF total metals @ 350 mV **	[2]
NiFe > CoFe > NiCo > Co > NiLa, NiCu > CoPi, Ni, NiCe<br><br>(electrodeposited oxide)	1 M NaOH	η_{OER} @ 10 mA cm^{-2}	[3]
NiFe > CoFe, NiLa > NiCo, NiCu, Ni, NiCe > Co > CoPi	“	ECSA-normalized by EIS (C_{DL}) @ non faradaic region (OCP ± 0.1 V) / by C_{DL} from different scan rates CV @ non faradaic region (OCP ± 0.1 V) ***	[3]
NiFe > NiCr > NiMn > Ni > NiCu > NiCo > NiZn<br><br>(co-precipitation at 80°C at constant pH)	0.1 M KOH	ECSA-normalized by pseudocapacitance measurements in non faradaic region (0.9-1.0 V_{RHE}) ***	[4]

*: intrinsic activity metrics

**: approaching a reasonable estimate for the intrinsic activity for very thin films, but not for thick catalyst layers.

***: intrinsic activity metrics. However, conductivity issues might arise with certain metal oxyhydroxides.[2] Extended non faradaic region are typically non available beyond the potential at which conductivity rises for many metal (oxy)hydroxides.

Supplementary Table 2. Co and Mn oxidation states from XANES analysis.

	Co edge position by integral method (eV)	Co oxidation state	Mn edge position by integral method (eV)	Mn oxidation state
$Co(OH)_2$	7718.59	2.02		
CoMn LDH	7719.90	2.59	6550.34	3.41
CoCo LDH	7719.83	2.56		
NiCo LDH	7721.06	3.10		
NiMn LDH			6549.68	3.14

Supplementary Table 3. Average nanoplate sizes by TEM analysis.

Samples	Average in-plane diameter (nm)	Thickness (nm)
NiFe LDH	404 ± 48	15
NiCo LDH	36 ± 6	6
NiMn LDH	108 ± 44	10
$Ni(OH)_2$	78 ± 9	10
CoFe LDH	113 ± 56	10
CoCo LDH	112 ± 87	7
CoMn LDH	96 ± 10	15
$Co(OH)_2$	2600	-

Supplementary Table 4. Relative stability of LDHs. Relative stability of the α- and γ-M_AM_B LDH, and β-M_AM_B oxyhydroxides (M_A= Ni, Co, and M_B= Ni, Co, Fe, Mn). ΔG_{form}, which is normalized by the total number of metal atoms in the unit cell (Supplementary Figure 9), is calculated with respective to the anhydrous (hydroxyl)oxides (brucite-like $M(OH)_2$, MOOH, and MO_2), water, and ions. For α-M_AM_B LDH, the reference ions are CO_3^{2-} that is in equilibrium with CO_2 in atmosphere (400 ppm). For γ-M_AM_B LDH, the reference ion is K^+ which has the standard reduction potentials 2.4 V lower than K_2O. See computational method for more details.

	ΔG_{form} (eV/M)		
M_AM_B	α-M_AM_B LDH	γ-M_AM_B LDH	β-$(M_{A0.75}M_{B0.25})OOH$
NiMn	-0.01	-0.01	-0.08
NiFe	-0.01	0.01	0.00
NiCo	-0.04	-0.13	0.07
NiNi	-0.04	-0.03	0
CoMn	0.00	-0.08	0.08
CoFe	0.01	-0.06	0.11
CoCo	-0.01	-0.11	0
CoNi	-0.19	-0.11	0.07

Supplementary Table 5. Intrinsic magnetic moment of Mn, Fe, Co and Ni in MO, M_2O_3 and MO_2 with 2+, 3+ and 4+ oxidation state. Those magnetic moments with ±0.1 μ_B margin are used to assign the oxidation of the metal atoms in more complex oxides and surface models.

	Magnetic moment (μ_B)		
Oxidation state	2+	3+	4+
Mn	4.6	3.8	3.1
Fe	3.7	4.1	3.3
Co	2.7	0.0	1.2
Ni	1.8	1.0	0.0

Supplementary Table 6. Atomic magnetic moments (μB) of α-M_AM_B LDHs. Corresponding tags of atoms ($M_{A/B}$-x) in Supplementary Figure 9. Atomic magnetic moments of Mn^{3+}, Fe^{3+}, Co^{3+}, Ni^{3+} are 3.9 μ_B, 4.2 μ_B, 0.0 μ_B and 1.0 μ_B, respectively. Atomic magnetic moments of Mn^{2+}, Fe^{2+}, Co^{2+}, Ni^{2+} are 4.6 μ_B, 3.7 μ_B, 2.7 μ_B and 1.8 μ_B, respectively.

M_AM_B	M_B-1	M_B-2	M_A-1	M_A-2	M_A-3	M_A-4	M_A-5	M_A-6
NiMn	3.9	3.9	1.8	1.8	1.8	1.8	1.8	1.8
NiFe	4.2	4.2	1.8	1.8	1.8	1.8	1.8	1.8
NiCo	0.0	0.0	1.8	1.8	1.8	1.8	1.8	1.8
NiNi	1.7	1.6	1.7	1.7	1.7	1.6	1.7	1.6
CoMn	3.9	3.9	2.7	2.7	2.7	2.7	2.7	2.7
CoFe	4.2	4.2	2.7	2.7	2.7	2.7	2.8	2.7
CoCo	0.0	0.0	2.7	2.7	2.7	2.7	2.7	2.7
CoNi	1.8	1.8	0.0	2.7	2.7	2.7	0.0	2.7

Supplementary Table 7. Atomic magnetic moments (μB) of γ-M_AM_B LDHs. Corresponding tags of atoms ($M_{A/B}$-x) in Supplementary Figure 9. Atomic magnetic moments of Mn^{4+}, Fe^{4+}, Co^{4+}, Ni^{4+} are 3.1 μ_B, 2.0 μ_B, 1.0 μ_B and 0.0 μ_B, respectively. Atomic magnetic moment of Fe^{5+} is 2.8 μ_B.

M_AM_B	M_B-1	M_B-2	M_A-1	M_A-2	M_A-3	M_A-4	M_A-5	M_A-6
NiMn	3.1	3.1	1.0	0.0	0.1	0.1	-0.9	0.1
NiFe	2.0	-2.0	-0.9	0.1	-0.1	0.9	0.1	-0.1
NiCo	0.0	0.0	0.0	0.0	0.0	0.0	0.0	0.0
NiNi	0.9	0.1	0.0	0.0	0.0	0.9	0.0	0.0
CoMn	3.1	3.1	1.2	0.0	1.2	0.0	1.2	1.2
CoFe	2.8	-2.8	1.1	0.0	0.0	1.1	0.0	0.0
CoCo	1.1	0.0	-1.1	-1.1	1.1	0.0	-1.1	1.1
CoNi	0.0	0.0	0.0	1.1	1.1	0.0	1.1	1.1

Supplementary Table 8. Atomic magnetic moments (μ_B) of β-M_AM_B oxyhydroxides. Corresponding tags of atoms ($M_{A/B}$-x) in Supplementary Figure 9.

M_AM_B	M_B-1	M_B-2	M_A-1	M_A-2	M_A-3	M_A-4	M_A-5	M_A-6
NiMn	3.2	-3.1	1.1	1.7	1.0	1.7	1.1	1.1
NiFe	3.5	-3.4	1.7	1.1	1.1	1.1	1.7	1.1
NiCo	-0.1	-0.1	1.2	1.3	1.1	1.3	1.2	1.1
NiNi	1.1	1.1	1.1	1.1	1.1	1.1	1.1	-1.0
CoMn	3.9	-3.9	0.0	0.0	0.0	0.0	0.0	0.0
CoFe	4.2	-4.2	0.0	0.0	0.0	0.0	0.0	0.0
CoCo	0.0	0.0	0.0	0.0	0.0	0.0	0.0	0.0
CoNi	1.1	-1.1	0.0	0.0	0.0	0.0	0.0	0.0

Supplementary Table 9. Atomic magnetic moments (μ_B) as a function of oxidation state. MO marks the idealized molecular orbital (MO) magnetic moment of metal atoms local at the octahedral coordination.

Oxidation	*M* (high-spin Mn)		*M* (low-spin Fe)		*M* (high-spin Fe)		*M* (high-spin Co)		*M* (low-spin Co)		*M* (low-spin Ni)	
State	MO	DFT	MO	DFT	MO	DFT	MO	DFT	MO	DFT	MO	DFT
2	5	4.6	0		4	3.7	3	2.7-2.8	1		2	1.8
3	4	3.9	1		5	4.2	4		0	0.0	1	0.9-1.0
4	3	3.1	2	2.0	4	3.4-3.5	5		1	1.1-1.2	0	0.0-0.1
5			3	2.8	3							

Supplementary Table 10. Thermodynamic correction used in the free energy calculations. The table shows the values for the zero point energies (ZPE), integrated heat capacity ($\delta H^{0 \rightarrow 298K}$), entropy at 298.15 K (TS@298K) and the solvation energies ($E_{solvation}$). For details see "Thermodynamic correction" in the Computational methods section.

	ZPE (eV)	$\delta H^{0 \rightarrow 298K}$ (eV)	TS@298K (eV)	$E_{solvation}$(eV)
H_2O	0.56	0.10	0.68	
H_2	0.27	0.09	0.41	
OOH*	0.47	0.05	0.08	-0.4
O*	0.07	0.03	0.05	0
OH*	0.39	0.03	0.03	-0.3

Supplementary Table 11. Reaction free energy (eV) and overpotential η(V) on the γ-LDH surfaces. The reaction free energies are from the oxidation of bridge OH to four consecutive proton coupled electron transfer steps (1) $4OH^{-}+OH^{*} \rightarrow 3OH^{-}+O^{*}+H_2O+e^{-}$ ($OH^{*}\rightarrow O^{*}$) (2) $4OH^{-}+OH^{*}\rightarrow 2OH^{-}+OOH^{*}+H_2O+2e^{-}$ ($OH^{*}\rightarrow OOH^{*}$), (3) $4OH^{-}+OH^{*}\rightarrow OH^{-}+O_2+2H_2O+3e^{-}$ ($OH^{*}\rightarrow {}^{*}+O_2$), (4) $4OH^{-}+OH^{*}\rightarrow O_2+2H_2O+4e^{-}+OH^{*}$ ($OH^{*}\rightarrow OH^{*}+O_2$). These are the data points in Figure 5.

	NiFe	CoFe	NiCo	NiNi	NiMn
OH*-->O*	1.68	1.58	1.76	1.90	1.85
OH*-->OOH*	3.02	2.94	2.82	2.98	2.89
OH*-->*+O_2	4.55	4.65	4.70	4.31	4.83
OH*-->OH*+O_2	4.92	4.92	4.92	4.92	4.92
η(V)	0.45	0.48	0.65	0.67	0.71

Supplementary Table 12. Adsorption free energy of intermediates on the γ-LDH surfaces. The adsorption free energy of OER intermediates OH*, O*, OOH* with the reference state of H_2O, and corresponding adsorption equations are calculated as $H_2O\rightarrow OH^{*}+0.5H_2$, $H_2O\rightarrow O^{*}+H_2$, and $2H_2O\rightarrow OOH^{*}+1.5H_2$, respectively.

	NiFe	CoFe	NiCo	NiNi	NiMn
H_2O -->OH*	0.37	0.27	0.22	0.61	0.09
H_2O -->O*	2.05	1.85	1.98	2.51	1.94
H_2O -->OOH*	3.38	3.21	3.04	3.59	2.98

Experimental Methods

Catalysts synthesis

The synthesis of NiFe LDH, CoFe LDH, β-$Ni(OH)_2$ and β-$Co(OH)_2$ were reported in ref. [5]. Briefly, NiFe LDH was obtained by homogeneous precipitation in an autoclave filled with a solution of nickel(II) acetate, iron(III) nitrate, water and dimethylformamide (DMF). CoFe LDH was prepared by co-precipitation of Co(II) acetate and iron(III) nitrate in water using 1 M potassium carbonate solution and purging the solution with N_2, followed by a solvothermal treatment in an autoclave. β-$Ni(OH)_2$ was synthesized using a two-step synthesis consisting of a precipitation step of nickel(II) acetate by 1 M KOH addition and a subsequent hydrothermal treatment in an autoclave. β-$Co(OH)_2$ was synthesized by homogeneous precipitation of cobalt(II) chloride in water with hexamethylentetramine (HMT) and under reflux in N_2 atmosphere.

NiCo LDH was synthesized using a two-step synthesis protocol, consisting of a coprecipitation method with a simultaneous oxidation of Co and a solvothermal treatment afterwards. Ni(II)$(OAc)_2$ and Co(II)Cl_2, in a Ni:Co ratio of 2:1, 3:1, 5:1 and 9:1, were dissolved in 40 ml H_2O. H_2O_2, in an equimolar ratio to Co(II), and 1 ml 1 M KOH were added simultaneously to precipitate the nanoparticles and oxidize Co(II) to Co(III). The solution was stirred for 4 hours, centrifuged at 8500 rpm for 15 min and poured away. The precipitate was redispersed in 16 ml DMF and 34 ml H_2O in an autoclave glass liner. The solution was heated in the autoclave for 16 hours at 130 °C, followed by 2 hours at 170 °C. The reaction solution was let cool down naturally. Afterwards, the washing protocol was applied. First, the reaction solution was centrifuged at 8500 rpm for 15 min. The solution was poured away and the nanoparticles were redispersed in 40 ml of a water/ethanol mixture (3:1). The solution was centrifuged again and poured away. The precipitate was twice redispersed in 30 ml H_2O, centrifuged and poured away. Afterwards the particles were freeze-dried overnight. A dark green powder was obtained.

NiMn LDH was synthesized similarly to NiCo LDH, with a two-step synthesis protocol. Ni(II)$(OAc)_2$ and Mn(II)Cl_2, in a Ni:Mn ratio of 2:1, 3:1, 5:1 and 9:1, were dissolved in 40 ml H_2O. H_2O_2, in an equimolar ratio to Mn(II), and 1 ml 1 M KOH were added simultaneously to precipitate the nanoparticles and oxidize Mn(II) to Mn(III). The solution was stirred for 4 hours, centrifuged at 8500 rpm for 15 min and poured away. The precipitate was redispersed in 16 ml DMF and 34 ml H_2O in an autoclave glass liner. The two-step temperature protocol already described above for NiCo LDH was applied, as well as the washing protocol. A brown powder was obtained.

Co(II)Co(III) LDH was synthesized by a similar process described by Ma et al.,[6] which consisted in a topochemical synthesis with selective oxidation of β-$Co(OH)_2$. Pink-colored β-$Co(OH)_2$ was synthesized by a similar process to that described above. As-prepared β-$Co(OH)_2$ (0.45 g) was dispersed and stirred with a magnetic stir bar in a Br_2 / CH_3CN solution (5 g/500 ml) in an air-tight flask at room temperature for 5 days. A blackish-brown product was collected by filtering and repeated washing with acetonitrile until the filtrate appeared colorless. The brown solid product was recovered by centrifuge (8500 rpm, 15 min). The precipitation was washed two times with an acetonitrile and two times with pure ethanol. The product was freeze-dried overnight.

CoMn LDH was synthesized by using a modified co-precipitation method that involved the oxidation of Mn^{2+} to Mn^{3+} during precipitation. Cobalt-II-chloride (183.84 mg, 0.78 mmol) and Manganese-II-chloride (77.58 mg, 0.39 mmol) were dissolved in 30 mL of ultra-pure water. The solution was purged

with N_2 for half an hour. Under continues stirring 33.3 µl of hydrogen peroxide and 200 µl of 1 M potassium carbonate solution were added together to the metal precursor solution. A blackish brown precipitation was formed. After an hour, the blackish brown solid product was recovered by centrifuge (8500 rpm, 15 min). The precipitation was washed two times with a water-ethanol mix (3:1) and two times with water. The product was freeze-dried overnight.

To increase the crystallite sizes the application of a solvothermal treatment to the CoMn LDH was further investigated, in analogy to CoFe LDH. The dry product was dispersed in 34 ml ultra-pure water and 16 ml DMF in an autoclave liner. The suspension was purged with Nitrogen for half an hour. The autoclave liner was inserted in the autoclave and the autoclave was purged with Nitrogen for an hour. For half an hour, the autoclave was heated at 60 °C with stirring to avoid boiling retardation. After that, the stirring was stopped and the suspension was heated for 16 hours at 60 °C. Then the temperature was raised to 80 °C for two hours. At last the autoclave was cooled down naturally for two hours. The blackish brown solid product was recovered by centrifuge (8500 rpm, 15 min), washed two times with a water-ethanol mix (3:1) and two times with water. The product was freeze-dried overnight. As shown in Supplementary Figure 1b and in contrast to the case of CoFe LDH, this treatment lead to formation of a spinel phase, and was therefore discarded.

Physico-chemical characterization

TEM images were acquired with a FEI TECNAI G2 20 S-TWIN transmission electron microscope with LaB6 cathode. The microscope operated at an accelerating voltage of 200 kV. For the preparation of the sample for TEM, a small amount of powder was dispersed in water and sonicated for 10 minutes. 10 µl of the solution were pipetted onto a copper mesh (400 Mesh) and dried for 10 minutes at 60° C. The TEM images were analyzed with ImageJ2x (2.1.5.0; National Institute of Health, USA). Inductively-coupled plasma optical emission spectroscopy (ICP-OES) data were obtained by a 715-ES-ICP analysis system (Varian). To prepare the ICP samples, 2 to 5 mg of the LDH powder was dissolved in a mixture of 2 ml nitric acid, 2 ml sulfuric acid and 6 ml hydrochloric acid and let to degas overnight. This solution was filled up to 50 ml with MillQ water. From this solution 5 ml was taken and 5 ml of MillQ water was added. An X-ray diffractometer (D8 Advance; Bruker) and an X-ray tube (KFL CU 2K) were used for XRD measurements. A small amount of the powdered samples was put on silicon plates. The samples were measured in a 2θ-range from 10° to 70° with a step size of 0.04° and from 6 to 8 s measurement time for each step. 40 kV accelerating voltage and 40 mA current were set at the X-ray tube.

Ink and catalyst layer preparation

Glassy carbon (GC) disks (5 mm diameter) were polished manually with a 5 µm and 0.05 µm micropolish alumina suspension for ~3 min each treatment using a nylon and microcloth polishing pads, respectively, before each catalyst coating. After polishing, the disks were cleaned three times by ultrasonication in water, acetone, and water and finally dried with a nitrogen flow. 800 µl isopropanol, 200 µl of pure water and 5 µl Nafion solution (5 wt%) was added to 1.97 mg of the catalyst powder. The solution was sonicated for 15 min with a 1/8 inch microtip sonifier. 10 µl of the ink was pipetted on the polished glassy carbon (GC) electrode surface and dried in an oven for 7 min at 60 °C. The loading of the catalyst on the GC electrode was 0.1 mg/cm².

RDE measurements

RDE electrochemical experiments were performed in a three-compartment glass cell with a rotating disk electrode and a potentiostat (Gamry) at room temperature. The working electrode consists in the catalyst coated GC electrode and is mounted in the head of a rotating shaft. A Pt-mesh and a Hydroflex reversible hydrogen electrode (RHE, Gaskatel) were used as counter electrode and reference

electrode, respectively. The counter electrode was placed in a compartment that was separated by a fine-porosity glass frit from the working electrode compartment and a Luggin capillary was used for the reference electrode. The reference electrode was controlled regularly in comparison to a home-made RHE consisting in a polycrystalline Pt disk under H_2 bubbling, but no corrections were necessary, confirming the proper functioning of the RHE. The calibration in respect to a RHE is a very important practice for reference electrodes,[7] and, specifically for RHE, precautious should be used in saturating the electrolyte with O_2 to avoid mixed potentials.[8] For this reason and to avoid oxygen reduction reaction (ORR) contributions in the cyclic voltammetry at low potentials, N_2 was used. The electrolytes were prepared with KOH pellets (semiconductor grade, 99.99% trace metals basis, Aldrich), and MilliQ water. The electrolyte was purified by using sacrificial $Co(OH)_2$ for the Co based samples and $Ni(OH)_2$ for the Ni based samples, according to the procedure described by Boettcher and coworkers[9] to remove Fe impurities. Only NiFe LDH was tested without further Fe purification of the electrolyte, since residual Ni ions in solution introduced by the purification can negatively influence its activity[10] and it already contains Fe. Briefly: First 2 g of $Ni(OAc)_2 \cdot 4H_2O$ were dissolved in 10 ml deionized water. By adding 20 ml 1 M KOH, $Ni(OH)_2$ precipitated. The solution was agitated and then centrifuged for 15 min at 8500 rpm. The particles were washed three times with a mixture of 20 ml H_2O and 2 ml 1 M KOH. Afterwards the solution was poured away and 50 ml 1 M KOH were added to the particles. The solution was agitated for at least 10 min before resting overnight. Then the mixture was centrifuged. The supernatant was decanted through a filter paper into a clean tube and stored until use. Similarly, $Co(OH)_2$ was precipitated from 1 g of $Co(NO_3)_3$ with 10 mL 0.1 M KOH and washed three times with 10 mL 1 M KOH, centrifugated and decantated. The so washed $Co(OH)_2$ was added to 50 mL 1 M KOH. The suspension was standing overnight in the fridge and centrifuged and decanted next day. For the measurements, the electrolyte was diluted to 0.1 M. All electrochemical measurements were carried out in N_2-saturated and rotation rate of 1600 rpm and repeated at least 3 times. The current density values reported are normalized by the geometric area (0.196 cm^2). Internal resistance (iR) correction was applied after the measurements by using the value of resistance obtained during electrochemical impedance spectroscopy (EIS). All the potentials reported are iR-corrected, unless otherwise stated. OER overpotentials were calculated by subtracting 1.23 to the potentials after post-measurement iR correction, referred to the RHE and corresponding to the specified geometric current density during the liner sweep voltammetry, i.e. 1 mA cm^{-2} or 10 mA cm^{-2}.

Electrochemical protocol

The sample is first introduced under open circuit potential (OCP) in the N_2 saturated electrolyte and chronoamperometry (CA) at OCP is applied to let the current stabilize. Then, potentiostatic EIS (PEIS) is applied at OCP to evaluate the resistance for the iR-correction. Following the PEIS, cyclic voltammetry (CV) was conducted as an activation treatment at the sweep rate of 50 mV s^{-1}. After the CV, linear sweep voltammetry (LSV) measurements were conducted at lower scan rate (1 mV s^{-1}) to evaluate the catalyst activity. PEIS was repeated to control the value of the resistance. Finally, after N_2 purging, another PEIS was conducted, but at 1.6 V_{RHE}, and used for ECSA evaluation.

The protocol is summarized in the following list, where the potential values are the applied potentials, not iR corrected.

1. OCP (60 s)
2. CA (E = OCP, 60 s)
3. PEIS (E = OCP, ν_1 = 100 kHz, ν_2 = 0.2 Hz, 10 points/decade)
4. CV (E_1 = 1 V_{RHE} for the Ni series and 0.6 V_{RHE} for the Co series, E_2 = 1.9 V_{RHE}, v = 50 mV s^{-1}, n = 50)
5. LSV (E_1 = 1.2 V_{RHE}, E_2 = 1.9 V_{RHE}, v = 1 mV s^{-1})

6. PEIS (E = OCP, v_1 = 100 kHz, v_2 = 0.2 Hz, 10 points/decade)
7. CA (E = OCP, 60 s)
8. 15 min N_2 purging
9. CA (E = 1.6 V_{RHE}, 60 s)
10. PEIS (E = 1.6 V_{RHE}, v_1 = 100 kHz, v_2 = 0.2 Hz, 10 points/decade)

Where E_1 = lowest potential, E_2 = highest potential, E = applied potential, v_1 = lowest frequency, v_2 = highest frequency, v = scan rate, n = number of cycles and t = time.

ECSA calculation

The ECSAs were evaluated by using the Gamry Echem Analyst data analysis software package to fit the Nyquist plots using the equivalent circuit model shown in Figure 3a and calculate the capacitances associated to the constant phase element (CPE_a) of the adsorbed OER intermediates. This method is not dependent on which OER intermediates, i.e. O*, OH*, OOH*, is covering the surface during the measurement, therefore the expression "OER intermediates" is intended as general. These species are in dynamic equilibrium and reversibly adsorbed on the OER active catalyst surface. The OER intermediate surface coverage is potential dependent, rises at the onset of OER and reaches a plateau at slightly higher potential of the OER onset, where the majority of surface sites are OER active.[11] The potential of 1.6 V_{RHE} is generally sufficient to reach this plateau, since at higher potentials bubble formation creates disturbance in the PEIS measurement, even though ideally it should be verified for each catalyst.[11] The capacitance of the OER adsorbed intermediates, C_a, can be decoupled from the double layer capacitance by using the equivalent circuit in Figure 3a. The impedance Z of a CPE is expressed by:

$$Z = \frac{1}{Y_0 \cdot (j \cdot \omega)^{\alpha}} \quad (1)$$

Where j is the imaginary unit, ω is the radial frequency, and Y_0 and the exponent α are the CPE parameters. Combining the impedance of the CPE and the resistance R in parallel, the following expression is obtained:

$$Z = \frac{R}{1 + R \cdot Y_0 \cdot (j \cdot \omega)^{\alpha}} \quad (2)$$

By analogy with the impedance of a Zarc element,[12] which contains the time constant τ,

$$Z = \frac{R}{1 + (j \cdot \omega \cdot \tau)^{\alpha}} \; , \quad (3)$$

it follows

$$R \cdot Y_0 = (\tau)^{\alpha} \; . \quad (4)$$

Finally, taking τ = RC, the following expression is derived:

$$C = \frac{(R \cdot Y_0)^{1/\alpha}}{R} . \quad (5)$$

The fit of the Nyquist plot provides the values of Y_0 and exponent α associated to the CPE_a and the corresponding parallel resistance R_a. These are then used by the Gamry Echem Analyst software to calculate the capacitance, C_a. Then the capacitances were normalized by the specific unit area capacitance (C_S) of 0.3 mF cm^{-2}.[13]

TOF calculations

The turnover frequency (TOF) values are calculated from the equations:

$$TOF = \frac{J}{4Fm^*} \quad (6)$$

$$m^* = L(\frac{M_{A,wt\%}}{M_{A,u}} + \frac{M_{B,wt\%}}{M_{B,u}}) \quad (7)$$

where J is the current density at the OER overpotential of 350 mV, as estimated from the LSV, F is the Faraday constant and m* is the mol of metal per cm^2. m* is obtained by multiplying the catalyst loading L (0.1 mg cm^{-2}) by the sum of the weight percentage of M_A ($M_{A,wt\%}$) and M_B ($M_{B,wt\%}$) divided by their respective atomic mass ($M_{A,u}$ and $M_{B,u}$, respectively). The weight percentages of M_A and M_B are obtained from ICP-OES measurements.

X-Ray absorption spectroscopy and calculation of oxidation states

X-ray absorption spectroscopy (XAS) of the brucite β-$Ni(OH)_2$ and β-$Co(OH)_2$ was performed at the µSpot beamline of Bessy II operated by the Helmholtz-Zentrum Berlin für Materialien und Energie GmbH, while XAS of the CoMn LDH, CoCo LDH, NiCo LDH, and NiMn LDH at the KMC-2 beamline of the Bessy II synchrotron. X-ray absorption spectra were recorded in the fluorescence mode using a scintillation detector. The samples were deposited by drop casting from an ink on carbon paper stripes that were previously cut from a gas diffusion layer (GDL) sheet (28BC, SIGRACET®, SGL group). The sample loading was 0.1 $mg_{catalyst}$ cm^{-2}. Each stripe was mounted on a holder and placed at 45° in respect to the incident beam and the detector. Reference metal foils were measured in transmission at the same time for energy alignment. Initial processing of the XAS data was performed using the program Athena.[14]

The numerical values for the Mn and Co edge positions were obtained by the integral method described in ref. [15], which is based on the integration of the edge region from $\mu = 0.15$ to 1.0. The oxidation states where then obtained by assuming a linear dependence of the oxidation state on the edge position. For Co edge position, the linear calibration from ref [15] was used, which consists in using a slope of 2.30 eV per oxidation-state unit (eV/ox) and axis intercept of 7713.94 eV. For the Mn edge positions, the slope and intercepts were obtained by the linear fit of the edge positions of reference MnO_2, α-MnO_2 and Mn_2O_3, assuming oxidation state +4 for the first two and of +3 for the Mn_2O_3. The result of linear regression was a slope of 2.48 ± 0.24 eV/ox and intercept of 6541.89 eV. The oxidation states calculated from the Mn and Co edges are reported in Supplementary Table 2. All the Ni edges overlap with the one of $Ni(OH)_2$ (Supplementary Figure 3), which is consistent with the expected Ni oxidation state of +2. For NiFe LDH and CoFe LDH we refer to our previous work, showing oxidation state of +2 for Ni and Co and +3 for both Fe edges.[5]

Computational Methods

Computational parameters

Self-consistent, periodic density functional theory (DFT) calculations were performed with the projected augmented wave (PAW) method,[16] as implemented in the Vienna Ab-initio Simulation Package (VASP).[17] To achieve a highly accurate description of strongly-correlated LDHs with weak interaction between layers, we employ an error alignment and error cancellation approach which was

developed in our recent work .[18] More specifically, this approach includes use of a Hubbard U[19] term to correct for self-interaction errors in 3d transition metal cations, a van der Waals functional (optPBE)[20] to describe the weak interaction that occurs between layered materials, and intercalated water and ions between layers, and use of a water based reference state for the calculations to avoid incorrect description of the gas phase O_2 reference with standard DFT-GGA methods. Its accuracy is demonstrated through redox and (de)hydration of Mn, Fe, Co and Ni based bulk oxides and (oxy)hydroxides with standard errors of 0.04 eV per reaction formula unit. Its performance on surface thermodynamics is also demonstrated in our recent work.[5, 21] U values, which are applied to d-orbitals of Fe, Co and Ni, are taken as 2.5 eV, 3.5 and 5.2 eV, respectively. For cell shape and volume relaxations of (hydroxy)oxide compounds, a cutoff energy of 500 eV is used for the planewave expansion. For the calculations that do not involve cell optimization a cutoff energy of 400 eV is employed. Monkhorst−Pack k-point grids are used for Brillouin zone integration. A (2×4×3) and a (4×4×2) k-point grid are employed for α- and γ-phase of LDH with R1 symmetry and β-phase, respectively. For the other bulk and surface calculations, equivalent or denser k-point grids are utilized. An orthorhombic box (14 × 15 × 16) Å3 and a single k-point (0.25, 0.25, 0.25) for the Brillouin zone sampling are used for gas phase species. The equilibrium geometries are obtained when the maximum atomic forces are smaller than 0.01 eV/Å and when a total energy convergence of 10^{-5} eV is achieved for the electronic self-consistent field loop.

Thermodynamic correction

The Gibbs free energy correction includes zero point energy (ZPE), enthalpy (δH) and entropy (TS), and solvation effect ($E_{solvation}$) corrections (listed in Supplementary Table 10) according to $G = E_{DFT} + ZPE + \delta H - TS + E_{solvation}$. Thermodynamic correction are used in the free energy calculations. Zero point energies are calculated with experimental vibrational data in Ref.[22], the integrated heat capacity ($\delta H^{0\rightarrow 298K}$) and entropy at 298.15 K are obtained from Ref.[23]. For water, the entropy is calculated at 0.035 bar through $S = S0 + k_B T ln(p/p^0)$ to derive the chemical potential of liquid water, because at this pressure gas-phase water is in equilibrium with liquid water at 298.15 K. The solvation energies ($E_{solvation}$) are evaluated through *ab initio* molecular dynamics simulations by filling the vacuum with liquid water with a thickness that is equivalent to 5 water bilayers. It worth noting that, in comparison with our previous work,[24] the solvation energy of surface OH is about 0.3 eV smaller, because of high OH density in the current case which leads to a very limited amount of hydrogen bonds formed with interface water.

Computational details of α-phase and γ-phase LDHs

The atomic-scale structures of the as-prepared phase and the active phase of NiFe and CoFe LDHs, i.e. the α-phase and the γ-phase with water molecules and ions intercalated between layers, have been identified in our previous work.[5] Here, using those structures as the starting point, we studied the stability of M_AM_B LDHs (M_A = Ni, Co, M_B = Mn, Fe, Co, and Ni) synthesized in the current work through DFT calculations.

To evaluate stability of α-phase LDH with respective to the component (hydroxy)oxides, water, and CO_2 in the atmosphere (400 ppm), we calculate the relative free energy of formation by considering the following process:

$$6A(OH)_2 + 2BOOH + CO_2(g) + 5H_2O(l) \rightarrow A_6B_2CO_3(OH)_{16} \cdot 4H_2O \quad (8)$$

Then the relative free energy of formation can be calculated with

$$\Delta G_{\text{form}} = \mu(\ A_6B_2CO_3(OH)_{16} \cdot 4H_2O) - 6\mu(A(OH)_2) - 2\mu(BOOH) - \mu(CO_2) - 5\mu(\ H_2O) \quad (9)$$

Where $A(OH)_2$ and BOOH are component (hydroxy)oxides with A^{2+} and B^{3+} oxidation state. The exothermic process indicates that the formation of LDHs from component (hydroxy)oxides are favourable in water under ambient condition.

To evaluate stability of γ-phase LDH with respective to the component (hydroxy)oxides, water, and ions (K^+ and OH^-), we calculate the relative free energy of formation by considering the following process:

$$6AO_2 + 2BOOH + 2H_2O(l) + 2K^+ + 2OH^- \rightarrow A_6B_2O_{16} \cdot 2K \cdot 4H_2O \quad (10)$$

Then the relative free energy of formation can be calculated with

$$\Delta G_{\text{form}} = \mu(\ A_6B_2O_{16} \cdot 2K \cdot 4H_2O) - 6\mu(\ AO_2) - 2\mu(BOOH) - 2\mu(\ H_2O) - 2\mu(K^+) - 2\mu(OH^-) \quad (11)$$

Where AO_2 and BOOH are component (hydroxy)oxides with A^{4+} and B^{3+} oxidation state.

Due to the challenge of description ions in standard DFT calculations, the chemical potential of K^+ and OH^- are replaced with

$$2\mu(K^+) - 2\mu(OH^-) = \mu(K_2O) + 2\mu(\ H_2O) + 4.8 \text{ eV} \quad (12)$$

where -4.8 eV is the reaction free energy of

$$K_2O + H_2O \rightarrow 2K^+ + 2OH^- \quad (13)$$

To evaluate stability of β-phase oxyhydroxides with respective to the component (hydroxy)oxides, we calculate the relative free energy of formation by considering the following process without consideration of the actually computational oxidation states:

$$6AOOH + 2BOOH \rightarrow A_6B_2(OOH)_8 \quad (14)$$

The thermodynamic potential for the oxidation of water to produce oxygen ($2H_2O \rightarrow O_2 + 4H^+ + 4e^-$) is 1.23 V at standard conditions (T = 298.15 K, P = 1 bar, pH 0), which is also 1.23 V vs. RHE (reversible hydrogen electrode in alkaline conditions. We consider the following four electron reaction paths of OER with the potential U vs. RHE in equation (15)-(18).

$$OH^* + OH^- \rightarrow O^* + H_2O + e^- \quad (15)$$

$$O^* + OH^- \rightarrow OOH^* + e^- \quad (16)$$

$$OOH^* + OH^- \rightarrow O_2 + V^* + H_2O + e^- \quad (17)$$

$$V^* + OH^- \rightarrow OH^* + e^- \quad (18)$$

The corresponding reaction free energies are calculated using the formula below.

$$\Delta G_1 = \Delta G_{O^*} - \Delta G_{OH^*} - eU \quad (19)$$

$$\Delta G_2 = \Delta G_{OOH^*} - \Delta G_{O^*} - eU \quad (20)$$

$$\Delta G_3 = \Delta G_{O2} - \Delta G_{OOH^*} - eU \quad (21)$$

$\Delta G_4 = \Delta G_{OH*} - eU$ (22)

ΔG_{OH*}, ΔG_{O*} and ΔG_{OOH*} are calculated through the following reactions.

$H_2O + * \rightarrow OH* + 0.5H_2$ (23)

$H_2O + * \rightarrow O* + H_2$ (24)

$2H_2O + * \rightarrow OOH* + 1.5H_2$ (25)

The sum of $\Delta G_1$to ΔG_4 is fixed to the negative of experimental Gibbs free energy of formation of an oxygen molecule (ΔG_{O2}) involving four electron transfer of 4 × 1.23 = 4.92 eV, as following:

$$\Delta G_1 + \Delta G_2 + \Delta G_3 + \Delta G_4 = 4.92 \text{ eV} \quad (26)$$

The theoretical overpotential is then readily defined as:

$$\eta = \max\{\Delta G_1, \Delta G_2, \Delta G_3, \Delta G_4\}/e - 1.23 \text{ [V]} \quad (27)$$

Determination of oxidation states

The oxidation states of metal atoms are mainly determined by their intrinsic magnetic moment. For some subtle cases, which will be discussed below, charge balance needs be taken into account. The intrinsic magnetic moments of an atom with different oxidation are first determined from their compounds with well-defined valance, e.g. MO, M_2O_3, MO_2 with 2+, 3+ and 4+ oxidation state. The intrinsic magnetic moments of Mn, Fe, Co and Ni, each metal in the 2+, 3+ and 4+ oxidation state, are given in the Supplementary Table 5. Then, the oxidation of the metal atoms in more complex oxides will be determined by comparing their magnetic moments with those in the Supplementary Table 5. For example, for α-NiFe LDHs, the magnetic moments for Ni and Fe are 1.8 and 4.2, respectively, which correspond to Ni^{2+} and Fe^{3+}, respectively. For γ-NiFe LDH (see Supplementary Table 7), while we can determine the Ni oxidation state based on Supplementary table 5, the Fe magnetic moment (2.0 μ_B) is not comparable to values in Supplementary Table 5. However, based on the charge balance, it is Fe^{4+} with low spin.

Additionally, for α-phase LDHs with the general formula of $(M(OH)_2)_8 \cdot CO_3^{2-} \cdot 4H_2O$, there are six M^{2+} ions and two M^{3+} ions in a unit cell. The atomic magnetic moments of the eight metals in the unit cell have been listed in Supplementary Table 6. We could determine that (1) the atomic magnetic moments of Mn^{2+}, Fe^{2+}, Co^{2+}, Ni^{2+} are 4.6 μ_B, 3.7 μ_B, 2.7 μ_B and 1.8 μ_B, respectively; (2) the atomic magnetic moments of Mn^{3+}, Fe^{3+}, Co^{3+}, Ni^{3+} are 3.9 μ_B, 4.2 μ_B, 0.0 μ_B and 1.0 μ_B, respectively. For the γ-phase LDHs with the general formula of $(MO_2)_8 \cdot 2K^+ \cdot 4H_2O$, there are six M^{4+} and two M^{3+} ions in a unit cell. The atomic magnetic moments of the eight metals in the unit cell have been listed in Supplementary Table 7, we could determine that (1) the atomic magnetic moments of Mn^{4+}, Fe^{4+}, Co^{4+}, Ni^{4+} are 3.1 μ_B, 2.0 μ_B, 1.0 μ_B and 0.0 μ_B, respectively; (2) the atomic magnetic moment of Fe^{5+} is 2.8 μ_B. For β-phase oxyhydroxides with the general formula of MOOH, the average oxidation state of the all ions is +3. The atomic magnetic moments of the eight metals in the unit cell have been listed in Supplementary Table 8. For β-phase NiFe oxyhydroxide, there are four Ni^{3+} (1.1 μ_B) and two Ni^{2+} (1.7 μ_B), hence, the two Fe ions are the +4 with the high-spin state of 3.4-3.5 μ_B , which is different from the low-spin state of 2.0 μ_B based on the idealized molecular orbital (MO) magnetic moment analysis of metal atoms local at the octahedral coordination. In summary, the atomic magnetic moments as a function of oxidation state are listed in Supplementary Table 9.

Supplementary Discussions

Supplementary discussion on the synthesis of crystalline M_AM_B LDHs

To obtain the targeted crystalline phases, synthesis conditions were optimized specifically for each catalyst. For example, the application to a mixture of Co- and Fe-based precursors of the established procedure for the synthesis of NiFe LDH[5, 25] resulted in an undesired spinel phase (Supplementary Figure 1a). Similarly, the exact same solvothermal treatment used to increase the crystallinity of CoFe LDH could not be applied to the CoMn LDH system, due to the undesired formation of $MnCo_2O_4$ spinel (Supplementary Figure 1b). Furthermore, in order to stabilize cobalt hydroxide in the LDH phase, Br^- was used as intercalated anions, which led to the preferential ordering in the interlayer region and the particular pattern in Figure 1 with enhanced (006) reflection, as reported in the literature.[26] The metal compositions also needed to be tuned to avoid a mixture of separated brucite and hydrotalcite-phases (Supplementary Figure 2), which would result in strong inhomogeneity and possible mask the electronic effect of the second metal due to the physical separation of the second metal atoms from the host metal atoms. Individually adapted synthesis protocols were developed that ensured the desired crystal phase. Only in one case, minor reflections indicated the presence of an undesired Mn_3O_4 phase in NiMn LDH at high Mn contents. In order to minimize a possible contribution to OER, [27]an intermediate Mn content was selected. Finally, it is worth to notice that the formation of the undoped Ni(II)Ni(III) LDH in the alpha LDH phase is strongly unfavourable, due to the instability of Ni in oxidation state 3+ in the unbiased state, and the formation of undoped Co(II)Co(III) LDH demands special conditions, *i.e.* Br^- anions. Therefore, apart from the CoCo LDH catalyst, the LDH phase must originate from the incorporation of the foreign second metal.

Supplementary Discussion on Tafel Slopes

Tafel analysis can provide valuable kinetics information. In particular, Tafel slopes may reveal insights in the catalytic mechanism by determining which step in the OER mechanism is the rate determining step (RDS). Some care must be adapted in deriving conclusions from Tafel slopes analysis and for the discussion about the challenges in the interpretations of Tafel slopes in OER we refer to ref. [3, 28]. Supplementary Figure 5a shows the Tafel plot obtained from the LSVs in the low potential range, where the behaviour is linear. The corresponding Tafel slopes are shown in Supplementary Figure 5b. NiFe, CoFe, CoCo, CoMn LDHs show similar Tafel slopes slightly above 40 mV dec^{-1}, between 43 and 46 mV dec^{-1}. $Co(OH)_2$ has a higher Tafel slope of ~56 ± 10 mV dec^{-1}, so within uncertainties closer to 60 mV dec^{-1}. Finally, NiCo LDH has a higher Tafel slope, ~73 mV dec^{-1}, while $Ni(OH)_2$ and NiMn LDH have similar Tafel slopes of ~95-100 mV dec^{-1}. We notice that the values for $Ni(OH)_2$, $Co(OH)_2$, NiFe LDH and CoFe LDH are consistent with the ones reported by Burke et al. for the electrodeposited analogue catalysts.[2, 9a] We note that the potential-dependent Tafel slope behavior often resulting in two linear slope potential regions for OER catalysts[28b, 29], as also being observed for the investigated LDH catalysts. As the high potential region was often narrow and difficult to analyze due to deviations from linearity, which are partly attributed to bubble formation but probably of multiple origins and difficult to discuss.[28b, 29] For this reason, for each catalyst the Tafel slope corresponding to the lowest potential range of the two linear regions was considered in our analysis. If we consider 43-46 mV dec^{-1} ~40 mV dec^{-1}, 56 mV dec^{-1} ~60 mV dec^{-1} and 99-103 mV dec^{-1} ~120 mV dec^{-1} and provided that we can apply the standard Tafel analysis, the RDS can be determined by using the formula[28b] for the Tafel slope, b:

$$b = \frac{59}{n' + n_r \beta} \quad (28)$$

Where n′ is the number of electrons transferred before the RDS, n_r is the number of electrons transfer in the RDS, β is the symmetry factor (usually taken as 0.5), the number of times that the RDS occurs during one complete cycle of the OER mechanism is assumed equal to 1 and b is in the unit of mV dec^{-1}. The application of the formula indicates that the rate determining step (RDS) is the second e^- transfer (n′=1, n_r=1) for the most active LDHs (NiFe, CoFe, CoCo, CoMn LDH), a chemical step after the first e^- transfer (n′=1, n_r=0) for $Co(OH)_2$ and the first e^- transfer (n′=0, n_r=1) for $Ni(OH)_2$ and NiMn LDH. The value for NiCo LDH of 74 mV dec^{-1} cannot be easily explained considering: 1) an integer number of e^- that transfer before the RDS, 2) an integer number of e^- that are involved in the RDS, 3) the typical value of 0.5 for the symmetry factor β and 4) the RDS occurring one time in the mechanism. However, considering deviations of the symmetry factor β from the value of 0.5, the Tafel slope of 74 mV dec^{-1} can be obtained by the first e^- transfer as RDS (n′=0, n_r=1) and β ~ 0.8. Deviations in Tafel slopes could result from mixture of phases. However, for NiCo LDH this do not seem the case based on the XRD.

References

[1] D. Y. Chung, P. P. Lopes, P. Farinazzo Bergamo Dias Martins, H. He, T. Kawaguchi, P. Zapol, H. You, D. Tripkovic, D. Strmcnik, Y. Zhu, S. Seifert, S. Lee, V. R. Stamenkovic, N. M. Markovic, *Nature Energy* **2020**, *5*, 222-230.

[2] M. S. Burke, S. H. Zou, L. J. Enman, J. E. Kellon, C. A. Gabor, E. Pledger, S. W. Boettcher, *J Phys Chem Lett* **2015**, *6*, 3737-3742.

[3] C. C. L. McCrory, S. H. Jung, J. C. Peters, T. F. Jaramillo, *Journal of the American Chemical Society* **2013**, *135*, 16977-16987.

[4] O. Diaz-Morales, I. Ledezma-Yanez, M. T. M. Koper, F. Calle-Vallejo, *Acs Catal* **2015**, *5*, 5380-5387.

[5] F. Dionigi, Z. Zeng, I. Sinev, T. Merzdorf, S. Deshpande, M. B. Lopez, S. Kunze, I. Zegkinoglou, H. Sarodnik, D. Fan, A. Bergmann, J. Drnec, J. F. d. Araujo, M. Gliech, D. Teschner, J. Zhu, W.-X. Li, J. Greeley, B. R. Cuenya, P. Strasser, *Nature Communications* **2020**, *11*, 2522.

[6] R. Ma, K. Takada, K. Fukuda, N. Iyi, Y. Bando, T. Sasaki, *Angew Chem Int Edit* **2008**, *47*, 86-89.

[7] S. Q. Niu, S. W. Li, Y. C. Du, X. J. Han, P. Xu, *Acs Energy Letters* **2020**, *5*, 1083-1087.

[8] A. C. Garcia, M. T. M. Koper, *Acs Catal* **2018**, *8*, 9359-9363.

[9] aM. S. Burke, M. G. Kast, L. Trotochaud, A. M. Smith, S. W. Boettcher, *J Am Chem Soc* **2015**, *137*, 3638-3648; bL. Trotochaud, S. L. Young, J. K. Ranney, S. W. Boettcher, *J Am Chem Soc* **2014**, *136*, 6744-6753.

[10] J. M. Ye, D. H. He, F. Li, Y. L. Li, J. B. He, *Chem Commun* **2018**, *54*, 10116-10119.

[11] S. Watzele, P. Hauenstein, Y. C. Liang, S. Xue, J. Fichtner, B. Garlyyev, D. Scieszka, F. Claude, F. Maillard, A. S. Bandarenka, *Acs Catal* **2019**, *9*, 9222-9230.

[12] J. R. Macdonald, R. L. Hurt, *J Electroanal Chem* **1986**, *200*, 69-82.

[13] S. Watzele, A. S. Bandarenka, *Electroanal* **2016**, *28*, 2394-2399.

[14] B. Ravel, M. Newville, *J Synchrotron Radiat* **2005**, *12*, 537-541.

[15] M. Risch, F. Ringleb, M. Kohlhoff, P. Bogdanoff, P. Chernev, I. Zaharieva, H. Dau, *Energ Environ Sci* **2015**, *8*, 661-674.

[16] aP. E. Blöchl, *Physical review B* **1994**, *50*, 17953; bG. Kresse, D. Joubert, *Physical review b* **1999**, *59*, 1758.

[17] G. Kresse, J. Furthmüller, *Physical review B* **1996**, *54*, 11169.

[18] Z. Zeng, M. K. Chan, Z.-J. Zhao, J. Kubal, D. Fan, J. Greeley, *The Journal of Physical Chemistry C* **2015**, *119*, 18177-18187.
[19] S. Dudarev, G. Botton, S. Savrasov, C. Humphreys, A. Sutton, *Physical Review B* **1998**, *57*, 1505.
[20] J. Klimeš, D. R. Bowler, A. Michaelides, *Physical Review B* **2011**, *83*, 195131.
[21] Z. Zeng, K.-C. Chang, J. Kubal, N. M. Markovic, J. Greeley, *Nature Energy* **2017**, *2*, 17070.
[22] aK. P. H. Huber, G, 1 ed., Van Nostrand Reinhold Co, **1979**; bT. Shimanouchi, NSRDS-NBS 39, **1972**.
[23] M. W. Chase, *NIST-JANAF Thermochemical Tables*, 4th ed., American Chemical Society, Woodbury, N.Y., **1998**.
[24] aZ. Zeng, J. Greeley, *Nano Energy* **2016**, *29*, 369-377; bL. Wang, W. Gao, Z. Liu, Z. Zeng, Y. Liu, M. Giroux, M. Chi, G. Wang, J. Greeley, X. Pan, C. Wang, *ACS Catal.* **2018**, 35-42.
[25] F. Dionigi, T. Reier, Z. Pawolek, M. Gliech, P. Strasser, *Chemsuschem* **2016**, *9*, 962-972.
[26] R. Z. Ma, J. B. Liang, X. H. Liu, T. Sasaki, *J Am Chem Soc* **2012**, *134*, 19915-19921.
[27] aJ. Melder, P. Bogdanoff, I. Zaharieva, S. Fiechter, H. Dau, P. Kurz, *Zeitschrift für Physikalische Chemie* **2020**, *234*, 925-978; bM. F. Tesch, S. A. Bonke, T. E. Jones, M. N. Shaker, J. Xiao, K. Skorupska, R. Mom, J. Melder, P. Kurz, A. Knop-Gericke, R. Schlögl, R. K. Hocking, A. N. Simonov, *Angew. Chem., Int. Ed.* **2019**, *58*, 3426-3432; cA. Ramírez, P. Hillebrand, D. Stellmach, M. M. May, P. Bogdanoff, S. Fiechter, *J. Phys. Chem. C* **2014**, *118*, 14073-14081.
[28] aM. W. Louie, A. T. Bell, *J Am Chem Soc* **2013**, *135*, 12329-12337; bR. L. Doyle, I. J. Godwin, M. P. Brandon, M. E. G. Lyons, *Phys Chem Chem Phys* **2013**, *15*, 13737-13783.
[29] K. S. Exner, H. Over, *Acs Catal* **2019**, *9*, 6755-6765.
